# Construction of a syntrophic *Pseudomonas putida* consortium with reciprocal substrate processing

**DOI:** 10.1093/synbio/ysaf012

**Published:** 2025-06-24

**Authors:** Barbora Burýšková, Jesús Miró-Bueno, Barbora Popelářová, Barbora Gavendová, Ángel Goñi-Moreno, Pavel Dvořák

**Affiliations:** Department of Experimental Biology (Section of Microbiology), Faculty of Science, Masaryk University, Kamenice 753/5, Brno 62500, Czech Republic; Systems Biology Department, Centro Nacional de Biotecnología, CSIC, Darwin 3, Madrid 28049, Spain; Department of Experimental Biology (Section of Microbiology), Faculty of Science, Masaryk University, Kamenice 753/5, Brno 62500, Czech Republic; Department of Experimental Biology (Section of Microbiology), Faculty of Science, Masaryk University, Kamenice 753/5, Brno 62500, Czech Republic; Systems Biology Department, Centro Nacional de Biotecnología, CSIC, Darwin 3, Madrid 28049, Spain; Department of Experimental Biology (Section of Microbiology), Faculty of Science, Masaryk University, Kamenice 753/5, Brno 62500, Czech Republic

**Keywords:** synthetic consortium, reciprocal substrate processing, *Pseudomonas putida*, disaccharides, mathematical model

## Abstract

Synthetic microbial consortia can leverage their expanded enzymatic reach to tackle biotechnological challenges too complex for single strains, such as biosynthesis of complex secondary metabolites or waste plant biomass degradation and valorisation. The benefit of metabolic cooperation comes with a catch—installing stable interactions between consortium members. Here, we established a mutualistic relationship in the synthetic consortium of *Pseudomonas putida* strains through reciprocal processing of two disaccharides—cellobiose and xylobiose—obtainable from lignocellulosic residues. Two strains were engineered to hydrolyse and metabolize these sugars: one grows on xylose and hydrolyses cellobiose to produce glucose, while the other grows on glucose and cleaves xylobiose to produce xylose. This specialization allows each strain to provide essential growth substrate to its partner, establishing a mutualistic interaction, which can be termed reciprocal substrate processing. Key enzymes from *Escherichia coli* (xylose isomerase pathway) and *Thermobifida fusca* (glycoside hydrolases) were introduced into *P. putida* to broaden its carbohydrate utilization capabilities and arranged in a way to instal the strain cross-dependency. A mathematical model of the consortium assisted in predicting the effects of substrate composition, strain ratios, and protein expression levels on population dynamics. Our results demonstrate that modulating extrinsic factors such as substrate concentration can help in balancing fitness disparities between the strains, but achieving this by altering intrinsic factors such as glycoside hydrolase expression levels is much more challenging. This study presents reciprocal substrate processing as a strategy for establishing an obligate dependency between strains in the engineered consortium and offers valuable insights into overcoming the challenges of fostering synthetic microbial cooperation.

## Graphical Abstract

**Graphical Abstract:**
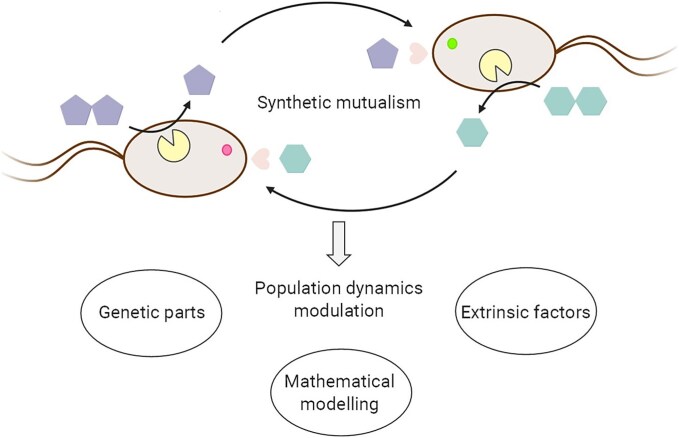

## Introduction

In nature, almost all microorganisms interact on a metabolic basis. This expands the enzymatic toolbox available for transforming their environment. The reach and abundance of microbes make them the geoengineers of new habitats, whose formation drives greater diversity, expanding the chemical conversion toolbox even further. Microorganisms are responsible for running whole biogeochemical cycles on our planet, and they achieve this through diversity and cooperation [1, 2].

As synthetic biologists aim to tackle increasingly complex biotechnological tasks [3, 4], they keep running into the wall of limitations set by tinkering with single-organism monocultures. In the past years, one of the approaches to solving the issue of monoculture limits became the engineering of whole microbial consortia [5–10]. These consist of multiple cooperating organisms, each bearing a part of an envisioned bioprocess to (i) complement each other and share the necessary burden [11–13], (ii) separate incompatible chemical reactions [14], (iii) support each other by removing waste [15], intermediates [16], or supplying coenzymes [17]. While the engineering limits are theoretically boundless due to the modularity of microbial consortia, establishing stable relationships between the individual consortium members is a recurring problem [18–20]. *De novo* interactions can be installed by quorum-sensing circuits that usually induce negative feedback loops in the consortium strains to maintain community balance [21, 22]. These, however, cause a high selective force for escapees that mutate or otherwise eliminate the threatening genetic components [23]. Another option is to instal a mutualistic relationship such as syntrophy, which relies on the consortium members benefiting from the presence of their consortial partners [24]. This approach is more in line with the inherent tendency of organisms to evolve and could lead to better long-term stability of the system [25–27]. Microbial syntrophy is a metabolic relationship between two or more microorganisms, where one partner’s growth relies on the nutrients, growth factors, or substrates supplied by the other(s) [1]. This often involves the exchange of metabolites or cross-feeding, allowing microbes to survive and function in specific niches. Cross-feeding typically occurs when one strain consumes a substrate and produces a downstream metabolite essential for the growth of another strain, such as an amino acid, a short-chain organic acid or fatty acid, or a vitamin [16, 17, 27, 28].

In this study, we aimed to explore the stabilizing potential of an approach that leverages cross-feeding with two substrates, which we term reciprocal substrate processing. We used the resilient saprophytic soil bacterium and robust biotechnological chassis *Pseudomonas putida* KT2440 as a host organism to test this concept [29, 30]. Due to its robustness and metabolic versatility, *P. putida* has emerged as an attractive microbial platform for processing lignocellulosic substrates, including sugars (hexoses and pentoses), organic acids, aromatic compounds, and even oligosaccharides [31–35]. In nature, the decomposition of plant biomass largely relies on microbial cooperation, making related processes ideal for showcasing the potential of synthetically established mutualistic relationships between consortial strains [36–39].

**Figure 1:**
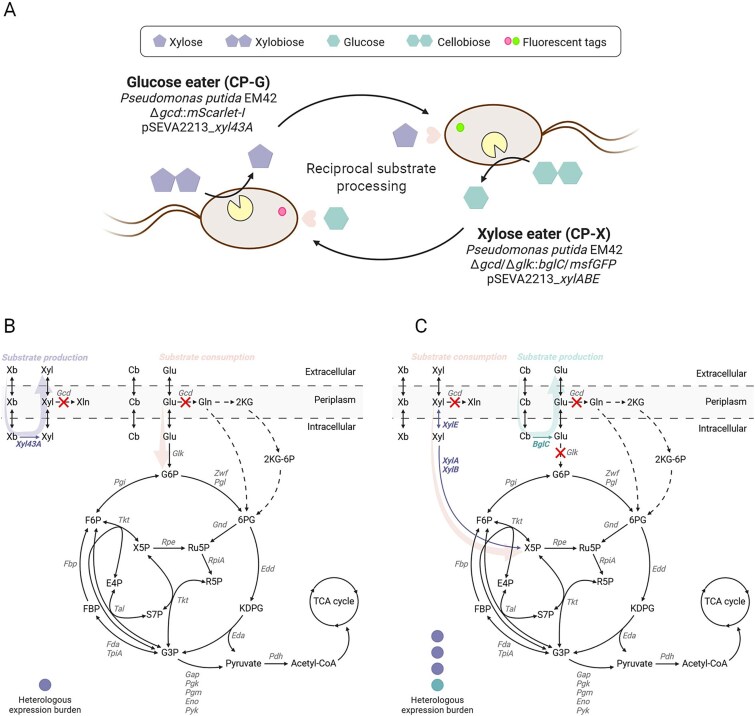
Consortium blueprint (**A**) and schematic illustration of sugar metabolism in the constructed *P. putida* strains CP-G (**B**) and CP-X (**C**). Heterologous enzymes and pathways are depicted in purple and teal. Deleted reactions are marked by a red cross. Native but vacant pathways are depicted by dotted arrows. The expected level of metabolic burden placed on the strains by heterologous expression is shown. Enzyme abbreviations: BglC β-glucosidase, Eda 2-keto-3-deoxy-6-phosphogluconate aldolase, Edd 6-phosphogluconate dehydratase, Eno phosphopyruvate hydratase, FBP fructose-1,6-bisphosphatase, Fda fructose-1,6-bisphosphate aldolase, gap glyceraldehyde-3-phosphate dehydrogenase, Gcd glucose dehydrogenase, Glk glucokinase, Gnd 6-phosphogluconate dehydrogenase, Pdh pyruvate dehydrogenase, Pgi glucose-6-phosphate isomerase, Pgk phosphoglycerate kinase, Pgl 6-phosphogluconolactonase, Pgm phosphoglycerate mutase, Pyk pyruvate kinase, Rpe ribulose-5-phosphate 3-epimerase, RpiA ribose-5-phosphate isomerase, Tal transaldolase, Tkt transketolase, TpiA triosephosphate isomerase, XylA xylose isomerase, XylB xylulokinase, XylE xylose/H^+^ symporter, Xyl43A β-xylosidase, Zwf glucose-6-phosphate dehydrogenase. Metabolite abbreviations: Cb cellobiose, E4P erythrose 4-phosphate, FBP fructose 1,6-bisphosphate, F6P fructose 6-phosphate, Gln gluconate, Glu glucose, G3P glyceraldehyde 3-phosphate, G6P glucose 6-phosphate, KDPG 2-keto-3-deoxy-6-phosphogluconate, 2KG 2-ketogluconate, 2KG-6P 2-ketogluconate 6-phosphate, 6PG 6-phosphogluconate, R5P ribose 5-phosphate, Ru5P ribulose 5-phosphate, S7P sedoheptulose 7-phosphate, Xb xylobiose, Xln xylonate, Xyl xylose, X5P xylulose 5-phosphate. Figures 1B and 1C were created with BioRender.com.

Building on previous achievements, we integrated engineered xylose and cellobiose metabolism in *P. putida* [31, 33, 40] with the newly introduced xylobiose catabolism to demonstrate the feasibility of the reciprocal substrate processing approach. *P. putida* was equipped with enzymes and transporters from *Escherichia coli* (XylA, XylB, XylE) and glycoside hydrolases from the cellulolytic actinomycete *Thermobifida fusca* (BglC, Xyl43A) to broaden its carbohydrate utilization capacity. Two *P. putida* strains were specialized to grow on either xylose (strain CP-X) or glucose (strain CP-G) monomers and cleave xylobiose (strain CP-G) or cellobiose (strain CP-X) disaccharides, respectively, to produce palatable substrate for their partner (Fig. 1). This strategy established an obligate dependency between the strains. Accompanied by a mathematical model describing the consortium behaviour, we further explored how modulating substrate composition, strain ratios, and protein expression can affect the population dynamics of such a system.

## Materials and methods

### Cultivation conditions

All strains used and prepared in this study are listed in Supplementary Table 1. For plasmid and strain construction, *E. coli* and *P. putida* strains were grown in lysogeny broth (LB) supplemented with appropriate antibiotics at 30°C and 37°C, respectively. For growth experiments, night cultures were grown 16 h in 2.5 ml of LB with antibiotics. These were centrifuged (all viable cells were centrifuged at 1500 g) and resuspended in M9 minimal medium, which consists of M9 salts (7 g/l Na_2_HPO_4_·7H_2_O, 3 g/l KH_2_PO_4_, 0.5 g/l NaCl and 1 g/l NH_4_Cl, pH 7.2), 2 mM MgSO_4_, 100 μm CaCl_2_, 3 ml/l trace element solution (0,3 g/l H_3_BO_3_, 0.05 g/l ZnCl_2_, 0.03 g/l MnCl_2_·4H_2_O, 0.2 g/l CoCl_2_, 0.01 g/l CuCl_2_·2H_2_O, 0.02 g/l NiCl_2_·6H_2_O, 0.03 g/l (NH_4_)_2_MoO_4_·2H_2_O and 0.03 g/l FeSO_4_). For the experiment, M9 minimal salts medium with specified carbon sources and antibiotics was used. Antibiotics were used at the following concentrations: ampicillin (Amp), 150 μg/ml for *E. coli* and 500 μg/ml for *P. putida*; kanamycin (Km), 50 μg/ml; streptomycin (Sm), 50 μg/ml; gentamicin (Gm), 10 μg/ml; tetracycline (Tet), 50 μg/ml; and chloramphenicol (Chl), 34 μg/ml.

### Plate reader growth experiments

Strains were precultured overnight in LB with appropriate antibiotics. The next day, the cultures were centrifuged (1500 g) and resuspended in M9 minimal medium. Growth assays were performed in Nunclon 96 Flat Bottom Transparent Polystyrene well plates (Thermo Fisher Scientific) containing 250 μl of M9 minimal medium supplemented with specified carbon sources (2 g/l unless stated otherwise) and antibiotics. Cultures were inoculated to an initial optical density (OD) of 0.05 as measured in cuvettes with a standard 1 cm path length. The well plate was covered with Breathe-Easy gas-permeable sealing membrane for microtiter plates (Diversified Biotech) and cultured in an Infinite M Plex microplate reader (Tecan). Continuous shaking was set to orbital with an amplitude of 2.5 mm, and each measurement was preceded by 10 s of linear shaking with an amplitude of 3 mm to avoid cell clumping. Fluorescence signal (bottom reading) and OD (OD_600nm_) were measured every half hour for 72 h. Fluorescence gain was determined from undiluted positive controls and then set manually and maintained throughout the study (60 for GFP, 90 for mScarlet-I).

For recalculation of single-strain fluorescence signal to single-strain OD in cocultures, continuous calibration curves (fitted by combined linear or polynomial functions) of each strain grown on respective monosaccharides were prepared in each experiment. The wells containing calibration samples served simultaneously as a control dataset for the recalculation. Considering the delay of mScarlet maturation, the OD of CP-G was calculated as the OD of the consortium minus the OD of CP-X, recounted from the reliable GFP signal.

### General cloning procedures

All plasmid constructs used and prepared in this study are listed in Supplementary Table 2. All plasmid constructs were built by standard restriction cloning or *in-vivo* cloning [41] using chemocompetent *E. coli* CC118 or *E. coli* DH5α λπ (prepared in-lab with Mix & Go! *E. coli* Transformation Kit and Buffer Set from ZYMO Research). Plasmid deoxyribonucleic acid (DNA) was isolated using E.Z.N.A. Plasmid DNA Mini Kit I (Omega Bio-Tek). DNA fragments were amplified by Q5 high-fidelity DNA polymerase according to the manufacturer’s instructions (New England BioLabs), purified using Monarch—polymerase chain reaction (PCR) & DNA Cleanup Kit or DNA Gel Extraction Kit (New England BioLabs), and the purity and concentration were determined by NanoDrop 2000 (Thermo Fisher Scientific). Colony PCR for strain verification and plasmid build confirmation was performed in a 10 μl volume using 2x DreamTaq Green PCR Master Mix (Thermo Scientific) with oligonucleotide primers (0.5 μm each) and the addition of Q5 High GC Enhancer in case of *P. putida* templates. All primers used were purchased by Merck and are listed in the Supplementary Table 3. Their annealing temperatures were calculated using NEB Tm Calculator (New England BioLabs). Standard sequencing was performed by Eurofins Genomics or SEQme Czech Republic, whole plasmid sequencing by Plasmidsaurus (using Nanopore technology). Positive clones were stored in cryogenic stocks (20% v/v glycerol in LB) at −70°C.

### Strain engineering

Plasmids were inserted into *P. putida* by electroporation (2.5 kV, 25 μF, 200 Ω) in a 2 mm gap width cuvette (Thermo Fisher Scientific) using GenePulser XcellTM (Bio-Rad). The preparation of electrocompetent *P. putida* cells and the electroporation procedure were performed as described by Wirth *et al.* [42]. Cells were refreshed in LB medium after electroporation for 2 h.

For direct genome editing (deletions, insertions, site-specific mutations), we used a two-step method based on homologous recombination (HR), where integrative plasmids are built to redesign specific genomic loci [43]. The plasmids cointegrate into the genome by HR and a second recombination is induced by double-strand breaks upon *in vivo* cleavage by I-SceI homing endonuclease from *Saccharomyces cerevisiae*. The resolution of the HR event has a theoretical outcome of 50% in favour of the wild type and 50% in favour of the edited sequence [43]. The protocol was optimized for *P. putida* by Martinéz-Garcia & Víctor de Lorenzo, where pEMG plasmid is used for cointegration and pSW-I for expression of I-SceI endonuclease [43]. Upgraded versions of this protocol use pSNW plasmids for cointegration, and self-curing pQURE for I-SceI expression [42, 44]. The glucokinase (PP_1011) deletion mutant Δ*glk* was built using pEMG. Insertion of degradation tags and deletion of N-terminal His tag was carried out according to the upgraded protocols. All genetic changes were checked by sequencing.

Tn7 insertions were carried out by mating. Four strains—*E. coli* CC118λπ pBG13/pBG13_mScarlet (bearing fluorescent protein genes as insertion cargo; donor strain), *E. coli* DH5α λπ pTnS-1 (strain leading transposase), *E. coli* pRK600 (helper strain), and a *P. putida* recipient strain—were mated by mixing equimolar amounts of cells in 10 mM MgSO_4_ and dropping the mix on an LB plate without antibiotics. The next day, a scoop of cells was resuspended in 10 mM MgSO_4_, diluted 100x, and 100 μl was spread on an M9 citrate (2 g/l) agar plate with 10 μg/ml Gm. The resulting *P. putida* transconjugants that exhibited fluorescence under blue light were selected for further work.

### Adaptive evolution of strain CP-X

The CP-X strain was grown in LB with Km overnight. The next day, a 50 ml Erlenmeyer Flask containing 6 ml of M9 minimal medium with Km and 2 g/l of xylose was inoculated to a starting OD of 0,05. The culture was passaged after 6 days (OD reached 0,23), and a second time after 3 days (OD 1,5). The final culture was harvested after 2 more days of growth in fresh medium (OD > 1) and stored at −70°C in a cryogenic stock (20% v/v glycerol in LB). The parental strain was renamed CP-X(pre) and the acclimated strain was named CP-X.

### Resting cell incubation for glycoside hydrolase activity testing

The conversion of disaccharide to monosaccharide by resting cells expressing β-glucosidase but unable to utilize glucose, and resting cells expressing β-xylosidase but unable to utilize xylose, was monitored by HPLC. The test strains CP-X and CP-G and negative control strains without glycoside hydrolase enzymes (ID24 and ID18) were grown as night cultures and these were used to inoculate 10 ml of fresh LB medium with appropriate antibiotics and the strains were cultured for another 6 h at 30°C and shaking at 200 rpm in Shaking Incubator NB-205, N-BIOTEK. The culture sample (1 ml) of OD 1 was taken to prepare a cell sample and a supernatant sample by centrifugation. The cells were washed with pure M9 medium and resuspended in M9 medium with appropriate antibiotics. One more ml of the test strain culture (OD 1 or equivalent) was used to prepare a lysate sample by lysing the cells with B-PER (Thermo Scientific). A sample of the negative control strain cells, prepared the same way as the cell sample of the test strain, was used as the negative control without enzyme. All samples were diluted to an identical volume of 1.5 ml and supplemented with 2 g/l of disaccharide and appropriate antibiotics. Two antibiotics were used in supernatant samples to inhibit any additional cell growth (Km and Chl). The samples were incubated overnight at 30°C with shaking at 200 rpm in a Shaking Incubator NB-205 (N-BIOTEK). The next day, all samples were centrifuged, and the resulting supernatants were prepared for HPLC analysis.

### HPLC analysis

The saccharide composition of growth experiment samples was analysed using the Agilent 1100 Series system (Agilent Technologies) equipped with a refractive index detector and Hi-Plex H, 7.7 x 300 mm, 8 μm HPLC column (Agilent Technologies). Collected bacterial cultures were centrifuged at 20 000 g for 10 min. The supernatant was filtered through 4 mm/0.45 μm LUT Syringe Filters (Labstore), diluted 1:1 with 50 mM H_2_SO_4_ in degassed Mili-Q water to stop any hydrolytic activity, and stored at −20°C in HPLC columns. Analysis was performed using the following conditions: mobile phase 5 mM H_2_SO_4_, mobile phase flow 0.5 ml/min, injection volume 20 μl, column temperature 65°C, RI detector temperature 55°C. Analyte concentrations were calculated from the area under the curve, related to respective analyte calibration curves prepared with pure chemicals purchased from Sigma-Aldrich (Merck).

### Whole genome sequencing

*Pseudomonas putida* EM42-derived strains were cultured in LB medium at 30°C till mid-exponential phase. Cells were collected and enzymatically treated: 10 ml of bacterial culture in mid-exponential phase cultivated in LB at 30°C was centrifuged at 3000 × g and 10°C for 10 min, washed with 5 ml of wash solution (10 mM Tris–HCl, 10 mM EDTA, 10 mM EGTA, 1 M NaCl of pH 7.5), and resuspended in Tris-EDTA (TE) buffer with achromopeptidase (1 000 U/ml, Sigma-Aldrich), lysozyme (5 mg/ml, Sigma-Aldrich), and RNase A (200 μg/ml, New England BioLabs) in a total volume of 500 μl, followed by incubation for 1–2 h at 37°C until lysis appeared. Then, 30 μl of 10% SDS and 5 μl of proteinase K (20 mg/ml, Sigma-Aldrich) were added, and the sample was incubated for 60 min at 50°C. Genomic DNA was extracted from cell lysate using the Genomic DNA Clean & Concentrator-25 kit (Zymo Research) according to the manufacturer’s instructions. For Oxford Nanopore sequencing, the library was prepared using the SQK-RBK004 Rapid Barcoding kit (Oxford Nanopore Technologies) according to the manufacturer’s instructions. The library was sequenced with a FLO-MIN106 flow cell (R9.4.1) in a MinION device controlled by MinKNOW software v.22.12.7 (Oxford Nanopore Technologies), which was also used for basecalling (super-accurate model with a minimum q-score threshold of 10), demultiplexing, and barcode trimming. Assembly of Nanopore reads was performed using Flye v.2.9.1 and Medaka consensus pipeline v1.7.2 (Oxford Nanopore). Genomic data was handled by Geneious Prime 2022.2.2 (Biomatters) and sequencing reads were mapped using the Geneious algorithm.

### SDS-page

For each sample, 2 ml of cells of OD 1 from night culture in LB was harvested by centrifugation at 4000 g at 4°C for 10 min and stored at −20°C after decanting the supernatant. Cell-free extracts were prepared using B-PER reagent (Thermo Scientific). Centrifugation at 14 000 rpm (Hettich Universal 320R) and 4°C for 15 min separated the cell-free extracts from cell debris. The cell-free extracts were stored at −20°C. Cell-free extract (4 μl) of each sample was combined with 2 μl of Sample loading buffer (5x; 1.2 ml Milli-Q H_2_O, 0.5 ml of 0,5 M Tris–HCl pH 6.8, 0.8 ml glycerol, 0.8 ml 10% SDS, 0.2 ml β-mercaptoethanol, a pinch of Bromphenol blue) and boiled at 95°C for 5 min. The samples, as well as 5 μl of Colour Protein Marker II (NZYtech), were then loaded onto a 12% separating, 4% stacking SDS-PAGE gel and run for 35 min. The gel was stained by Quick Coomassie stain (Serva) for 1 h, destained in dH_2_O overnight, and scanned.

### BglC and Xyl43A activity assays in cell lysates and culture supernatants

For the activity assays with cell lysates, 2 ml of CP-G or CP-X cells of OD 1 from night culture in LB was harvested by centrifugation at 2500 g at room temperature for 10 min. Cell pellets were lysed by adding 100 μl of BugBuster Protein Extraction Reagent with 1 μl of Lysonase Bioprocessing Reagent (both from Merck Millipore) for 15 min at room temperature. β-glucosidase and β-xylosidase activity were measured using synthetic substrates *p*-nitrophenyl-β-D-glucopyranoside (pNPG) and *p*-nitrophenyl-β-D-xylopyranoside (pNXG), respectively (both from Sigma-Aldrich). Reaction mixture of total volume = 600 μl contained 550 μl of 100 mM sodium phosphate buffer (pH 7.0), 30 μl of pNPG or pNXG (final conc. 5 mM), and 20 μl of 50-fold diluted cell lysate. Reaction was started by adding cell lysate to the mixture of buffer and substrate in Eppendorf tube, pre-incubated 10 min at 37°C. Samples (120 μl) were withdrawn periodically and the reaction was terminated by adding 80 μl of 1 M Na_2_CO_3_. Absorbance of the mixture in the wells of microtiter plate was measured at 405 nm using Infinite M Plex microplate reader (Tecan) and activity was calculated using calibration curve prepared with *p*-nitrophenol standard (Sigma-Aldrich). Enzyme activities in culture supernatants were measured correspondingly with 40 μl of non-diluted culture supernatant in 620 μl of reaction mixture. 1 unit (U) of enzyme activity corresponds to 1 μmol of substrate (pNPG or pNXG) converted by enzyme per 1 min. Total protein concentrations in cell lysates and culture supernatants were measured by Bradford reagent (Sigma-Aldrich) according to the manufacturer’s instructions. Crystalline bovine serum albumin (Sigma-Aldrich) was used as a protein standard. Activities were measured in three biological replicates.

### Fluorescence microscopy

Cell samples were centrifuged (1500 g, 3 min), washed with M9 minimal medium, and fixed with 8% formaldehyde and 0.25% glutaraldehyde solution (1:1 v/v) for 30 min in Cellview cell culture slides (polystyrene, 75/25 mm, glass bottom; Greiner). The fixed samples were washed with MilliQ water and covered with Vectashield Antifade Mounting Medium (Vector Laboratories). The cells were visualized using ZEISS Elyra 7 with lattice SIM mounted with the ZEISS objective Plan-Apochromat 63x/1.4 Oil DIC M27 and captured in pixel size 60 nm for channels 488 nm and 561 nm in 3D. Data was processed with Zeiss software SIM2 and visualized as orthogonal projection.

### Statistics

The number of repeated experiments or biological replicates is specified in figure and table legends. The mean values and corresponding standard deviations are presented.

## Results and discussion

### Design of the consortium

The design of our consortium was guided by a few key principles. We wanted each strain to specialize in utilizing one type of sugar substrate—glucose for strain CP-G and the *P. putida* non-native substrate xylose for strain CP-X. Such an approach prevents potential sequential substrate uptake or crosstalk caused by carbon catabolite repression (CCR) [45, 46] and it allowed us to build two independent populations as the basis for our consortium. We chose the disaccharide versions of each sugar—cellobiose and xylobiose—as the primary substrates that can be obtained from lignocellulosic biomass [33, 47]. Heterologous expression of the glycoside hydrolases needed for cleaving the two disaccharides presented us with a unique opportunity to set up a mutual relationship between the two populations. Each strain would express the glycoside hydrolase, cleaving the substrate to be assimilated by the second strain. This reciprocal substrate processing made both strains dependent on one another in a form of syntrophy (Fig. 1) [1, 27].

Substrate specificity was ensured by the lack of a xylose utilization pathway in wild-type *P. putida*, which became the Glucose eater (CP-G). Heterologous plasmid-based expression of *xylABE* genes from *E. coli* encoding the xylose isomerase pathway and xylose transporter (pSEVA2213_*xylABE*) was the basis of the Xylose eater (CP-X), together with a deletion of the glucokinase gene *glk* (PP_1011), disabling the growth on glucose [31]. Both strains harboured a deletion of the periplasmic glucose dehydrogenase gene *gcd* (PP_1444) to block the conversion of xylose to the dead-end product xylonate (Fig. 1) [31]. To distinguish the strains, fluorescent tags preceded by a translational coupler were introduced into the chromosome *via* the mini-Tn7 transposon system from Zobel *et al.* [48] targeting the genes into the *att*Tn7 site for stable basal expression. The Xylose eater was tagged with msfGFP while the Glucose eater was tagged with mScarlet-I [49]. The reciprocal substrate processing was established by the heterologous expression of glycoside hydrolases from the cellulolytic actinomycete *T. fusca*. The Glucose eater produced Xyl43A β-xylosidase (Tfu_1616) from pSEVA2213_*xyl43A*, whereas the Xylose eater produced BglC β-glucosidase (Tfu_0937), whose gene was genome-integrated *via* the mini-Tn5 transposon system [43]. Both enzymes were expressed with an N′-terminal His tag and should be located intracellularly [50, 51]. The setup of the strains was expected to place a higher metabolic burden on the Xylose eater due to the load of heterologous enzymes expressed in addition to metabolizing a non-native substrate (Fig. 1) [10]. Nevertheless, the reciprocal substrate processing was expected to establish obligate cooperative dependency.

### Components testing and initial consortium characterisation

The consortium members held their respective substrate specificities. When first constructed, the Xylose eater showed limited growth in liquid medium with its respective substrate xylose (Supplementary Fig. 1). Since the phenotype was confirmed on solid medium (not shown), we decided to culture the strain in liquid medium with xylose until growth commenced. We then inoculated fresh xylose medium twice, and the time needed for the culture to reach visible growth plummeted from six to three to two days. The initial growth stagnation was first thought to be caused by the *glk* deletion affecting the regulation of neighbouring genes encoding enzymes of the EDEMP (reactions of the Entner-Doudoroff, Embden-Meyerhof-Parnas, and pentose phosphate pathways) cycle [52]. But after the template (CP-Xpre) and improved strain (CP-X) were sequenced, a large genome multiplication (275 kb) was found in CP-X (2.6 times higher coverage than in CP-Xpre) framed by IS4 family transposase ISPpu8 (Supplementary Table 4). This multiplication included transaldolase *tal* [PP_2168] involved in the pentose phosphate pathway (PPP), which is strongly engaged in xylose utilization, and glyceraldehyde-3-phosphate dehydrogenase *gapB* [PP_2149], funnelling carbon out of the EDEMP cycle towards the TCA cycle. Multiplication of the same genetic region occurred in the adaptive laboratory evolution of *P. putida* EM42 on xylose in the study by Dvořák *et al.* [40], where the effect of *tal* overexpression on xylose utilization was confirmed by reverse engineering. The recurrence of this adaptive genome rearrangement shows how quickly *P. putida* can adjust its metabolic capacity to different directions of carbon influx.

**Figure 2:**
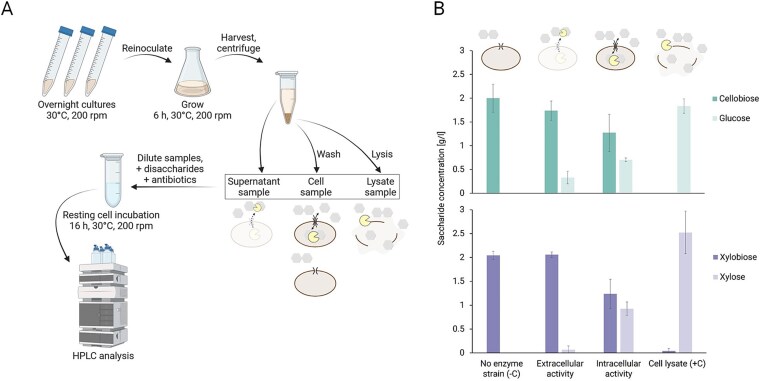
Resting cell experiment verifying disaccharide cleavage by heterologous glycoside hydrolases and transport through the cellular envelope. (**A**) Workflow of the experiment. Details can be found in the methods section. (**B**) Conversion of disaccharides to monosaccharides by consortium strain cells (intracellular activity), their culture supernatants (extracellular activity), or lysates (cell lysate +C), and negative controls (No enzyme strain -C, ID18 and ID24) after 16 h of incubation with 2 g/l of disaccharides. BglC activity is depicted in the upper graph, Xyl43A activity is depicted in the lower graph. Columns represent means ± standard deviations calculated from four biological replicates from two independent experiments (*n* = 4). Figure 2A was created with BioRender.com.

In both consortium strains, the glycoside hydrolases—β-glucosidase BglC and β-xylosidase Xyl43A—were well expressed. BglC made up to 12.3% of the total protein in CP-X and CP-G contained 26% of Xyl43A as visualized by SDS-PAGE (Supplementary Fig. 2). We then tested their activity by observing disaccharide cleavage in resting cells using HPLC analysis (Fig. 2). We confirmed that the intracellular hydrolytic activity of both enzymes, together with minor background activity of molecules that leached out of the cells (visible especially for CP-X and BglC), efficiently produces monosaccharides. Both strains are thus capable of supplying substrate for their consortial counterpart. Since the majority of enzymatic activity is intracellular, this suggests that a transport mechanism for cellobiose and xylobiose is present in *P. putida* EM42 (Fig. 2). The transport of cellobiose into the cytoplasm of *P. putida* has already been reported, unlike the transport of the non-native disaccharide xylobiose [31, 33]. The effect of transport mechanisms on microbial metabolism and ensuing bioengineering efforts is an understudied and thus an interesting topic for further exploration [53].

**Figure 3:**
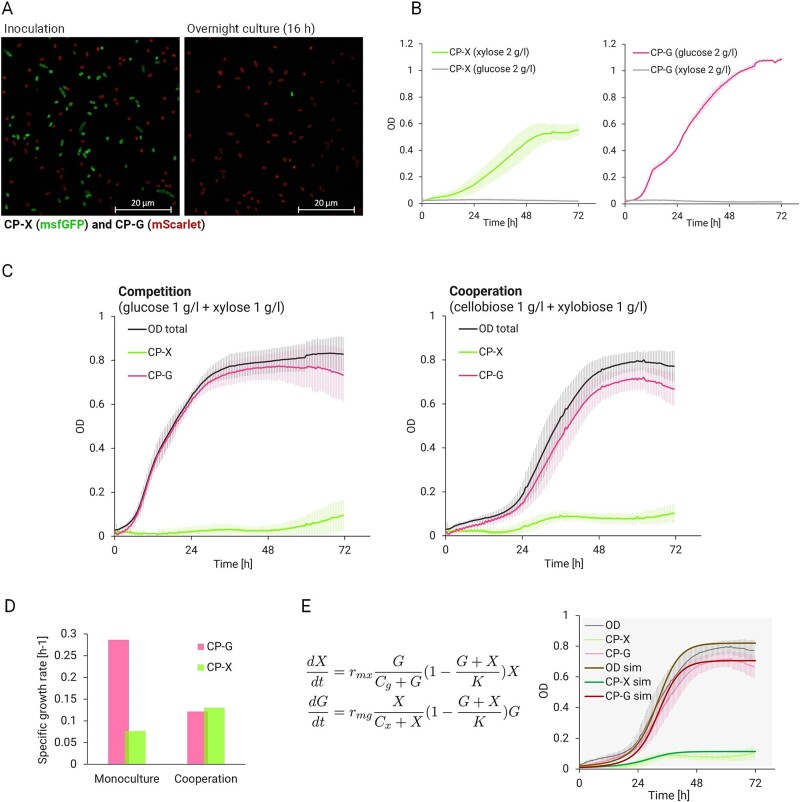
Characterisation of consortium strains. (**A**) Micrographs of basal fluorescence recorded with ZEISS Elyra 7. CP-G tagged by mScarlet and CP-X tagged by msfGFP in a 1:1 strain ratio at the beginning (left) and after overnight cultivation (16 h, right) of the consortium in complex LB medium. (**B**) Monoculture growth in 96-well plate format on respective monosaccharides. Data are shown as means ± standard deviations from several independent experiments and multiple biological replicates (CP-X on xylose *n* = 13, CP-X on glucose *n* = 9; CP-G on glucose *n* = 7, CP-G on xylose *n* = 8). (**C**) Cocultivation in 96-well plate format on monosaccharides (competition; left) and on disaccharides (cooperation; right). Data are shown as means ± standard deviations from nine independent experiments and at least 18 biological replicates (*n* ≥ 18). In (**D**) the changes in maximum specific growth rate between monocultures (from B) and cooperation (C, right) are shown. The values are calculated from the depicted average growth curves. (**E**) Mathematical model of the consortium. The behaviour of the consortium is described by two differential equations (left). The variables *X* and *G* represent the OD of the strains CP-X and CP-G. The parameters *r_mx_* and *r_mg_* are the *maximum specific growth rates* for *X* and *G*. The factor *G/(C_g_ + G)* represents the transformation of xylobiose into xylose by strain CP-G, while the factor *X/(C_x_ + X)* represents the transformation of cellobiose into glucose by strain CP-X. The parameters *C_x_* and *C_g_* account for the amount of glycoside hydrolases. Specifically, *C_x_* and *C_g_* are inversely proportional to the BglC and Xyl43A enzymes. Hence, when BglC (or Xyl43A) increases, *C_x_* (or *C_g_*) decreases, and the factor *X/(C_x_ + X)* (or *G/(C_g_ + G)*) approaches its maximum value of 1 more quickly as X (or G) increases. The factor *(1—(G + X)/K)* indicates the fraction of the free carrying capacity of the medium, where parameter *K*, known as the *carrying capacity*, depends on available resources. The values of these parameters were calculated by fitting the model to the growth curves obtained from the cooperating consortium (right). The initial conditions are *X_0_* = 0.01 and *G_0_* = 0.01.

Basal fluorescent protein expression enabled easy strain distinction under the microscope as well as during growth experiments in plate reader format (Fig. 3). We compared continual calibration during growth on monosaccharides with calibration using LB-grown overnight cultures and decided to use continual calibration on monosaccharides throughout the project since it better reflected the OD (Supplementary Fig. 3). The slower maturation of mScarlet expressed by CP-G led to calibration bias, and while it served well in reporting the general state of strain ratios, we decided to use the measured OD of all cells subtracted by the reliable GFP signal of CP-X cells for graphical depiction of CP-G cells in cocultivations.

Both members of the consortium were then cocultivated on monosaccharides (1 g/l xylose +1 g/l glucose) and disaccharides (1 g/l xylobiose +1 g/l cellobiose). The different cultivation conditions enabled the observation of competitive behaviour on monosaccharides or in rich LB medium (Fig. 3A) and cooperative dependency on disaccharides (Fig. 3C). On monosaccharides, no carbon cross-feeding was needed. Each strain was consuming its cognate monosaccharide and competing with its consortial partner for other nutrients and oxygen. Thus, the growth rate differences dictated the outcome where strain CP-G overgrew strain CP-X. On the disaccharides, the dependency of the two strains was indicated by the long lag phase of the whole consortium (~20 h) corresponding to the lag phase of CP-X alone, and by the simultaneous transition of both strains into the exponential phase (Fig. 3C, Supplementary Fig. 4). Since the dependence is enabled by the activity of the heterologously expressed glycoside hydrolases, the onset of the delayed exponential phase of CP-G was thus thought to mark the accumulation of enzyme and resulting monosaccharide in the culture. The established relationship also altered the growth rates of the individual strains when grown in a cooperating consortium compared to monocultures grown on monosaccharides. Whereas the maximal specific growth rate of CP-X benefited from the cooperation, the maximal specific growth rate of CP-G decreased (Fig. 3D). This shows that although the growth of the monocultures is not comparable, the engineered relationship establishes dependence of both strains in reciprocal substrate supply, which affects the growth pattern of the consortium members (Supplementary Fig. 4).

### Mathematical modelling of consortium behaviour

The gathered cocultivation data was used to build a mathematical model describing the behaviour of the consortium when grown on disaccharides. The model consists of two differential equations that describe the logistic growth of each strain (Fig. 3E). This modelling approach not only allows us to represent the cross-feeding interactions between the two bacterial strains but also informs design decisions aimed at improving the consortium’s performance. The model builds on established principles in microbial dynamics, which explore logistic growth models for continuous population dynamics [21, 54–56].

The accuracy of our model was validated by predicting the growth of the consortium strains cocultivated on disaccharides and comparing it to the measured data (Fig. 3E). We manually fitted the parameters *r_mx_*, *r_mg_*, *C_x_*, *C_g_*, and *K* of the model using the total OD data from the experiment and the CP-X OD data from the calibration. Specifically, the values of these parameters were calculated from the growth curves obtained during the consortium cultivation on disaccharides (Fig. 3C, right). Then, we plotted the OD of the strain CP-G and checked that the predicted OD of CP-G was similar to the one obtained using the equation OD_total_ = OD_x_ + OD_g_. The model was then employed to search for cultivation variables (substrate ratios, inoculation ratios) that, when adjusted, would balance the growth of the two consortial strains. To make predictions for cultivation variables, we used scaling factors represented by the symbol *α*. Specifically, *G/(C_g_ + G)* is scaled based on the initial amount of xylobiose (*α_Dg_*), and *X/(C_x_ + X)* is scaled based on the initial amount of cellobiose (*α_Dx_*). In this way, we could account for the effect of using different substrate ratios and make new predictions, as shown below. The parameter *r_mx_* is scaled by the burden due to BglC expression and degradation (*α_Bx_*). The parameter *C_x_* is scaled according to the amount of BglC (*α_Ex_*). Additionally, the value of *K* is scaled by both the initial amount of disaccharides and the amount of the BglC enzyme, represented by *α_K_*, where *α_K_ = min{α_Ex_, α_Dx_, α_Dg_}*. Consequently, *K* is scaled by the minimum scaling factor—i.e. the most restrictive one. With these adjustments, the final form of the two differential equations shown in Fig. 3E is:


$$ \frac{dX}{dt}=\frac{r_{mx}}{\alpha_{Bx}}\ \frac{\alpha_{Dg}G}{C_g+G}\left(1-\frac{G+X}{\alpha_KK}\ \right)X $$

$$ \frac{dG}{dt}={r}_{mg}\frac{\alpha_{Dx}X}{C_x/{\alpha}_{Ex}+X}\left(1-\frac{G+X}{\alpha_KK}\ \right)G $$

For the consortium experiment in Fig. 3E the scaling factors *α_Dx_*, *α_Dg_*, *α_Bx_*, and *α_Ex_* were all set to 1 and the parameter values were *r_mx_* = 0.12 h^−1^, *r_mg_* = 0.34 h^−1^, *C_x_* = 0.04, *C_y_* = 0.02, and *K* = 0.82. The benefit of the mathematical model developed in this study is its ability to explore the system’s potential beyond what is immediately observable in the lab. The process began with experimental work, followed by calibrating the model based on these experiments. This calibrated model then allowed us to conduct *in silico* studies to explore the various possibilities within the system, such as optimizing cultivation conditions and predicting system behaviour under different scenarios.

### Mathematical model enables searching for optimal cultivation conditions

**Figure 4:**
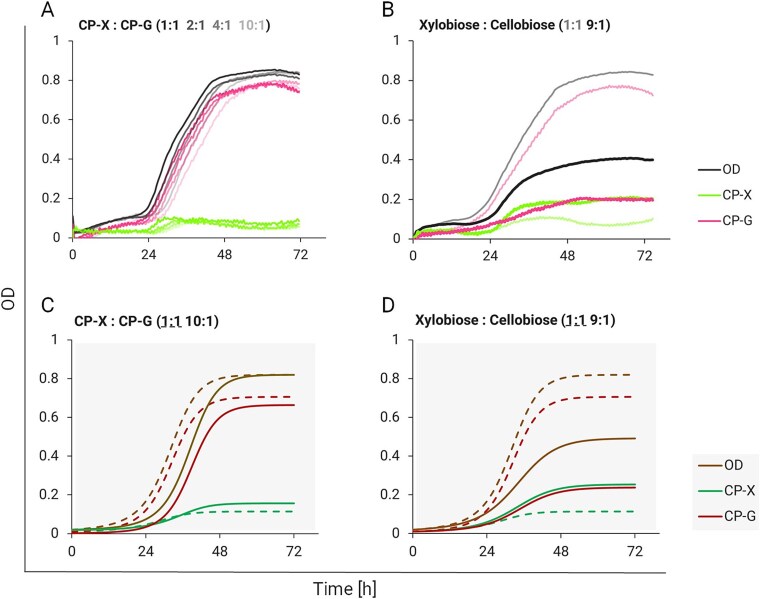
Consortium cocultivation on disaccharides (1 + 1 g/l) with different strain inoculation ratios (**A**) and substrate ratios (total sugar concentration always 2 g/l) (**B**) in 96-well plate format. Data are shown as means from two *(n* = 2) biological replicates. The line saturation corresponds with the saturation of the specified conditions. Error bars are omitted for clarity. The bottom graphs show respective model-predicted outcomes. (**C**) Simulation of the consortium with initial conditions *X_0_* = 0.0182 and *G_0_* = 0.0018. (**D**) Simulation of the consortium with high xylobiose (*α_Dg_* = 1.4) and low cellobiose (*α_Dx_* = 0.6). Dashed lines represent the starting point of the consortium with unaltered strain or substrate ratios, for reference.

A possible means of balancing the consortium when strain growth rates are unequal is choosing inoculation ratios that favour the slower-growing strain [20]. We thus repeated the cocultivation with inoculation ratios 2:1, 4:1, and 10:1 in favour of CP-X. None of the cultivations favouring CP-X balanced the strain growth curves (Fig. 4A). Additionally, a higher inoculation ratio in favour of CP-X only prolonged the lag phase of both strains. The CP-G overgrowth trend stayed and shows that the population dynamics pattern is stable, as observed for other obligate cross-feeding consortia [28]. Since an advantage in numbers did not help CP-X to achieve better growth, as predicted by the model (Fig. 4C), we tried to cultivate the consortium on different ratios of disaccharides, which could give us more insight into the consortium dynamics and another opportunity to challenge the predictions of our mathematical model.

Altering the substrate ratio balanced the growth of our consortium strains. When limiting the Glucose eater with substrate (9 parts xylobiose: 1 part cellobiose, 2 g/l in total), the resulting ratio of CP-X and CP-G in the consortium was 1:1 (Fig. 4B). However, the final OD of the consortium was two times lower compared to the consortium grown on equal amounts of both disaccharides. Taken together, our data show in line with the mathematical prediction (Fig. 4D) that while inoculation ratios do not balance the growth of the cocultivated strains, the substrate ratios do. This could be leveraged in a continuous cultivation system by formulating the inflow of substrates in a way that would maintain consortium balance [57, 58]. On the other hand, asymmetric growth could also be leveraged by adding an anabolic pathway, and thus burden, to the faster growing less burdened strain CP-G. This could be another way to stabilize the consortium by tightening the growth rates of both strains.

### Searching for bottlenecks

**Figure 5:**
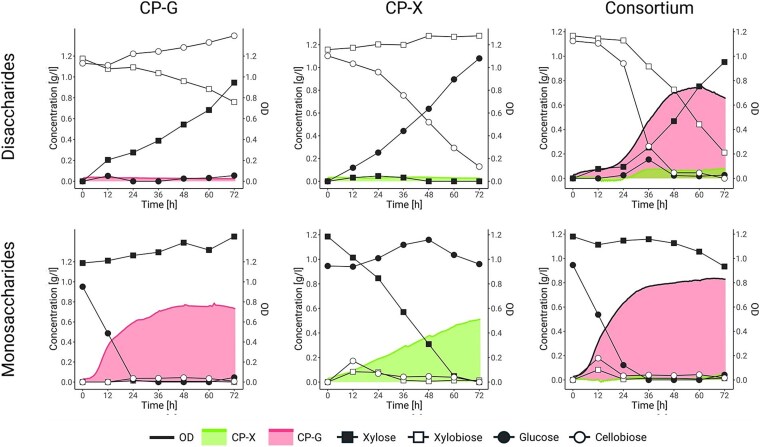
Consortium and monoculture cultivations on disaccharides and monosaccharides in 96-well plate format with saccharide concentration monitoring by HPLC. OD is depicted as an area mapped on the right y-axis, whereas sugar concentrations are depicted with line graphs mapped on the left y-axis. Data are shown as means of at least three biological replicates from two independent experiments (*n* ≥ 3). (error bars are omitted for better clarity; the same figure containing error bars is available in supplementary information as Supplementary Fig. 5). Note that slight evaporation of culture volume towards the end of the experiment causes sample concentration, which makes the amount of abundant sugars seem to rise.

To see how fast the saccharide production and consumption are in real-time, we repeated the cultivation of the consortium and individual consortium strains on monosaccharides and disaccharides and included regular sampling for HPLC analysis (Fig. 5 and Supplementary Fig. 5).

The experiment was carried out in a 96-well plate format for direct comparison with earlier cultivations. Multiple technical replicates were used to ensure sufficient sampling volumes. As expected, CP-X and CP-G individually only consumed their respective monosaccharide substrates. Interestingly, the growth patterns slightly differed from ones obtained on single monosaccharides (Fig. 3B), pointing towards altered sugar assimilation regulation when two types of sugar substrates are present. When grown individually on disaccharides (Fig. 5), no growth occurred, yet the inoculation biomass itself (250 μl of OD 0.05) contained enough glycoside hydrolases to convert the majority of the respective disaccharide to monosaccharide. This confirmed that the enzymes are well expressed (Supplementary Fig. 2) and active, and should not pose a bottleneck in strain cross-feeding. Interestingly, the conversion rate of xylobiose by CP-G (0.006 ± 0.001 g/l·h, calculated from disaccharide depletion; Fig. 5) was two-fold lower than the conversion rate of cellobiose by CP-X (0.014 ± 0.003 g/l·h). This was in contrast to the two-fold higher expression of Xyl43A in CP-G compared to BglC in CP-X (Supplementary Fig. 2). A two-fold higher specific activity of Xyl43A measured in CP-G cell lysates (2.86 ± 0.18 U/mg), compared to the specific activity of BglC in CP-X cell lysates (1.27 ± 0.06 U/mg), correlated with the higher expression of the former enzyme and suggested that xylobiose transport into the cell may be the limiting factor. Moreover, enzyme activity measurements in culture supernatants confirmed moderate BglC activity (3.33 ± 0.96 U/l) and only minimal Xyl43A activity (0.61 ± 0.12 U/l). We propose that both the poorer transport of xylobiose into CP-G and the leakage of BglC from CP-X may contribute to the observed differences in oligosaccharide conversion rates.

Cocultivation of the consortium strains on monosaccharides showed the rapid consumption of glucose by the CP-G strain and a minimal decrease in xylose consumed by the negligible growth of the CP-X strain (Fig. 5). A temporary rise of disaccharides (around 12 h) is possibly caused by the reverse activity of the glycoside hydrolase enzymes in the presence of a high product-to-substrate ratio [59]. Cocultivation on disaccharides shows that even though both glycoside hydrolases are active, conversion of cellobiose to glucose is faster when both strains enter exponential phase (Consortium—Disaccharides**;** see incline in cellobiose and xylobiose concentration between 24 and 48 h). At the end of the experiment, the majority of xylose is still available in the growth medium. The fitter CP-G strain arguably depletes other nutrients or oxygen in the medium, disabling further growth of CP-X.

### Tightening the mutualistic dependence with degradation tags

**Figure 6:**
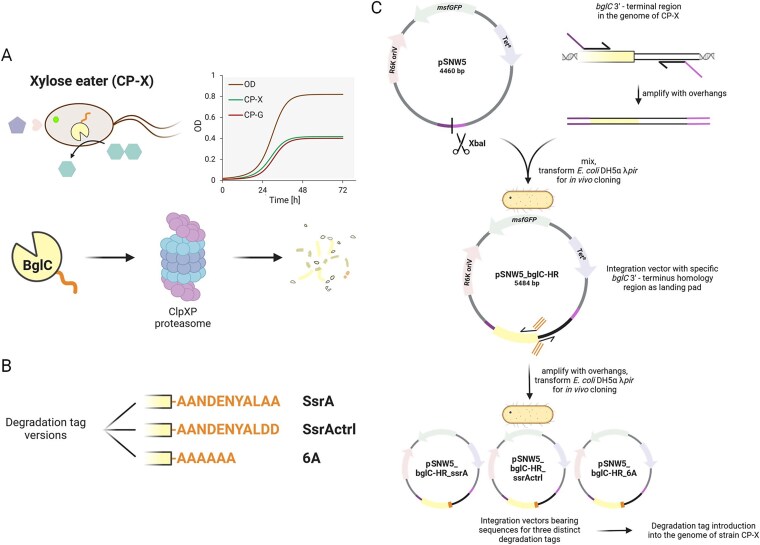
Reduction of BglC excess in CP-X by degradation tags. (**A**) Schematic illustration of the degradation-tagged BglC fate with a simulation of the expected effect on the consortium dynamics (for the simulation, *C_x_* was set to 0.08 and *r_mx_* to 0.22 h^−1^). (**B**) Amino acid sequences of the three degradation tags. (**C**) Cloning workflow for targeted addition of the degradation tags into the CP-X genome. Details can be found in the methods section. Figure was created with BioRender.com.

After gathering the information from the first Design-Build-Test-Learn cycle, for the second round, we decided to tighten the consortium members’ relationship. The major molecular parts that enable the mutualistic relationship are the glycoside hydrolases, and from the HPLC analysis of the cocultivation medium we knew that β-glucosidase produced by CP-X hydrolysed disaccharides faster than β-xylosidase from CP-G (Fig. 5, Consortium—Disaccharides). We thus decided to link β-glucosidase (BglC) activity more tightly to the growth of CP-X by increasing its degradation rate to prevent its accumulation. This would limit the rate at which glucose substrate for the CP-G strain would be produced. A simulation by the mathematical model supported our idea that decreasing the amount of BglC could help establish a more balanced growth of the consortial strains (Fig. 6A). For this, we introduced sequences to the 3′ end of the *bglC* gene that would translate into C-terminal degrons and mimic natural proteolytic signalling recognized by the native ClpXP protease complex [60]. We chose a *Pseudomonas*-specific SsrA degradation tag (AANDENYALAA) together with its two-amino acid substitution variant used as a control by Keiler *et al.* (AANDENYALDD) [61–63] and a poly-A degradation tag (AAAAAA), representing a conserved ribosome-associated quality control mechanism mediated by RcqH (Fig. 6B) [64]. The tags were introduced in front of the STOP codon of the *bglC* gene in the chromosome of strain CP-X using pSNW2-based integration vectors constructed by *in-vivo* cloning (Fig. 6C) [42].

**Figure 7:**
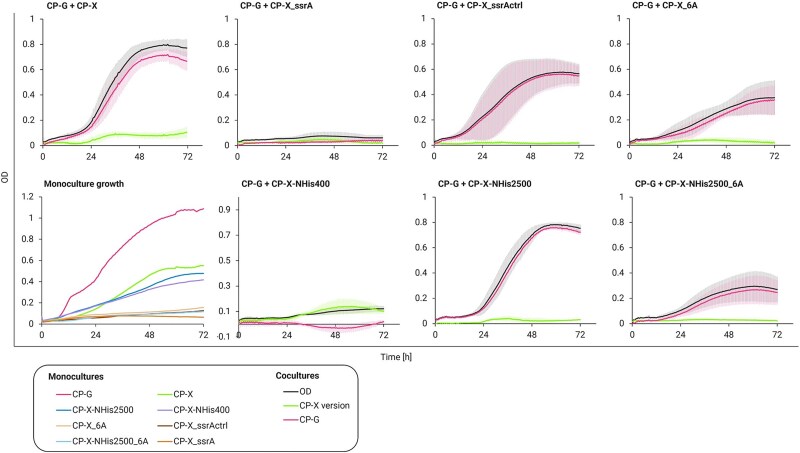
Consortium cooperation in 96-well plate format on disaccharides (1 + 1 g/l) with CP-X strain variants representing different BglC expression and degradation patterns, leading to modulated relationships of both consortial strains. Monoculture growth on xylose (2 g/l; CP-X) or glucose (2 g/l, CP-G) shows individual strain burden (bottom left) that affects the consortium growth as well. Experimental data are shown as means ± standard deviations from at least three (*n* ≥ 3) biological replicates from two independent experiments.

The CP-X strain versions with degradation-tagged BglC were first grown individually to test their growth (Fig. 7). All degradation-tagged BglC strains grew worse than the template strain. We argue that this was caused by the highly efficient targeting of their β-glucosidase (12.3% of cellular protein; Supplementary Fig. 2) to ATP (adenosine triphosphate)-dependent proteasomes. We confirmed this by SDS-PAGE analysis, which showed that in the CP-X_ssrA strain, all BglC was degraded (0% of cellular protein; Supplementary Fig. 2) and in CP-X_6A, 75% of it was degraded (3.1% of cellular protein; Supplementary Fig. 2). Yet, why the growth of CP-X_ssrActrl with only a 6.5% BglC decrease was impaired is not clear (11.5% of cellular protein; Supplementary Fig. 2). A possible explanation could come from the work of Fritze *et al.*, who found that SspB, a mediator protein involved in SsrA-based protein degradation, could recognize the amino acid sequence of the _ssrActrl control tag [65]. This bond could make SspB unavailable for the native functions of the ribosome rescue pathway and protein recycling, and thus slow down cellular growth even without the costly BglC degradation.

Next, the strains were cocultivated as consortia on disaccharides and consortia with BglC degradation were compared to the template consortium (Fig. 7). The high BglC degradation activity of the ssrA tag completely disabled the growth of CP-G and of the whole consortium. Nevertheless, the relationship and thus the fate of CP-G and CP-X strains were tightly linked in this consortium. Microscopic imaging revealed an interesting finding, as this consortium formed clusters of cells after cocultivation (Supplementary Fig. 6). To test whether this effect was caused by the strong mutual dependency, in which proximity of the cells could be beneficial [66], we had to rule out stress from this experiment since it causes cell clumping on its own. In *P. putida*, the stress-response sigma factor σRpoS activates the expression of adhesin LapF involved in cell–cell interaction [67, 68]. Hence, the consortium was also cultured in pure M9 medium without a carbon source as a stress control. Both the experiment and control samples were cultured in a 96-well plate format for 24 h for microscopy imaging, where a 5 x 5 grid of 60 x 60 μm pictures was taken, and the cell clusters of this area were counted. The clump ratio of CP-X only: mixed: CP-G only was ~1:2:1 in the control sample and 1:4:1 in the experiment sample (Supplementary Fig. 6). Although a larger dataset should be examined, this preliminary experiment suggests that the cells are actively attaching to their cognate strain and spatially organizing as a response to the engineered relationship [69]. The work of Pignon *et al.*, [69] describes metabolite uptake rate and leakage rate as two factors influencing the consortium structure and composition. In the case of CP-X_ssrA, the (glucose) leakage rate is very low and might lead to CP-G growth closer to the available glucose source, promoting CP-X—CP-G specific clumping. We need to note, however, that our consortial strains do not have a flagellum [29], and thus their native aggregation mechanisms might be disturbed [70].

Both the 6A and ssrActrl degradation tags resulted in a lower final OD of the consortium, with most biomass still belonging to CP-G (Fig. 7). The lag phase differed in length from the template consortium, perhaps due to stress and cell lysis of CP-X, enabling more BglC to escape into the medium and produce glucose faster. Overall, the effect of both the ssrA and 6A degradation tags was confirmed, yet none of the variants led to a viable tightening of the strain growth curves. We hypothesized that the high production (RBS, ribosome binding site) strength and theoretical translation rate of 13 018 a.u., leading to 12.3% of total cellular protein in SDS-PAGE Supplementary Fig. 2) and subsequent degradation of BglC leads to an unnecessary burden on the Xylose eater and *bglC* expression must be tuned down before the addition of degradation tags for these to have any positive effect.

We thus constructed CP-X variants with weakened expression of *bglC*. The N-terminal histidine tag sequence influences the 5’UTR of the *bglC* gene and increases theoretical translation strength significantly, as determined with the Salis Lab RBS calculator [71]. We thus used its removal as a tool to lower translation strength and alleviate some metabolic burden by lowering the production of BglC targeted for degradation in ATP-dependent proteasomes. Firstly, we only removed the N-terminal His tag, resulting in strain CP-X-NHis2500 with a *bglC* RBS strength of 2589 a.u. (theoretical translation rate). Secondly, we removed the N-terminal His tag and additionally switched the original RBS to an *in silico*-designed RBS sequence (Salis Lab RBS calculator—design mode) [71] for an even lower RBS strength of 417 a.u. resulting in strain CP-X-NHis400. These two CP-X strains with lowered BglC expression resulted in very different consortium outcomes. In the case of the CP-X-NHis2500, the consortium grew almost identically to the template consortium, pointing to a persisting excess of BglC enzyme. On the other hand, strain CP-X-NHis400 did not allow the consortium to grow much at all. The RBS strength reduction in strain CP-X-NHis400 was considerable, resulting in no BglC expression (0% of cellular protein; Supplementary Fig. 2), which explains the nonexistent CP-G growth. The majority of the low OD was attributed to CP-X, which possibly utilized the small amounts of xylose that were produced by residual Xyl43A enzyme in the inoculated CP-G cells (see CP-G growth on disaccharides in Fig. 5). We also constructed one strain in which we combined the two approaches by lowering BglC expression in the CP-X_6A, resulting in CP-X-NHis2500_6A. This combination still led to a high burden placed on the Xylose eater, and the consortium grew to an even lower OD than the CP-X_6A strain consortium.

These outcomes have shown us that finetuning the glycoside hydrolase expression is an easy way to modulate the relationship of our consortium. We did, however, not find the sweet spot that would allow both strains to grow at a similar rate while reaching a high OD. It is possible that exploring more of the design space would lead to the envisioned outcome [72]. In hindsight, we recognize degradation tags to be a very good tool, but not the best one for a low-burden fix of unbalanced growth rates of consortium strains. Finetuning the relationship bolts´ expression (glycoside hydrolases in our case) or timing the expression by placing the genes into naturally occurring operons with desired expression characteristics could be an economical way to modulate the population dynamics. Alternatively, an anabolic pathway placed in the fast-growing CP-G strain could have the same effect with added functionality.

Employing the developed mathematical model to make predictions in the right direction is another possibility. For the CP-X strain variants with weakened expression and/or degradation of *bglC*, our model simulations predict an overall OD imbalance between CP-X and CP-G (Supplementary Fig. 7), a trend consistent with experimental observations (Fig. 7). However, the quantitative predictions diverge from the experimental data; the model typically shows a higher OD for CP-X and a lower OD for CP-G than was experimentally measured. These results highlight the model’s current limitations in accurately accounting for the intrinsic metabolic burden experienced by these engineered CP-X variants. To enhance the model’s predictive power for such scenarios, and thus its utility in guiding the search for an optimal balance, future improvements could incorporate parameters that explicitly represent metabolic load, [73] for instance, by accounting for the energetic cost of heterologous enzyme synthesis and degradation.

It is also possible that the observed BglC leakage and extracellular activity have an impact on cross-feeding dynamics. To investigate this possibility, we extended our model to include parameters for such extracellular enzymatic conversions (Supplementary Fig. 8). The simulations suggest that high extracellular BglC activity enhances CP-G growth and reduces CP-X abundance, accelerating the consortium’s approach to its carrying capacity. On the other hand, potentially high extracellular Xyl43A activity would boost CP-X growth, allowing it to achieve a final abundance comparable to CP-G. This scenario leads to a more balanced population distribution within the consortium without altering the overall carrying capacity. Such simulations indicate that extracellular activity could indeed alter the final population balance of the individual strains, offering another possible means of tuning consortia with reciprocal substrate processing.

## Conclusion

In this study, we engineered two *P. putida* strains to reciprocally process two disaccharides—cellobiose and xylobiose—and exchange the resulting monosaccharides—glucose and xylose—to test our hypothesis that this strategy could establish a stable mutualistic relationship in a synthetic microbial consortium. We divided the catabolic routes for each sugar type—pentose (xylose, metabolized by strain CP-X) and hexose (glucose, metabolized by strain CP-G)—but placed the cognate glycoside hydrolases in the opposite strains to create obligate dependencies [1, 27]. The glycoside hydrolases (BglC and Xyl43A) represented the bolts of this relationship, and adjusting their expression or turnover (*via* degradation tags) was an easy way to modulate the population dynamics of the two consortium strains. Throughout this exploration, we confirmed how much burden is placed on cells through genetic engineering and that for any envisioned bioprocess, the ‘energetic price’ of circuits and pathways must be kept to a minimum [10, 73]. Besides modulating the population dynamics of the system intrinsically (through genetic parts), extrinsic factors like substrate ratios or inoculation ratios can also lead to desired changes in the system. The mathematical model, based on differential equations representing the logistic growth of each strain, allowed us to explore the effects of these extrinsic factors. By accurately predicting growth outcomes, the model confirmed that modulating substrate ratios was an effective strategy for balancing the growth of the two strains in coculture. The differences in growth rates could also be leveraged for the addition of new functions to the less-burdened strain. The modular nature of synthetic consortia gives researchers multiple degrees of freedom in designing the catabolic and anabolic pathways, but also the biotechnological process itself [74, 75]. The advantages of modularity and complexity, however, come with their own set of challenges. In our synthetic consortium, we successfully established a mutualistic relationship through reciprocal substrate processing. The population balance was, however, tilted, as dictated by strain burden. We managed to level this balance by modulating extrinsic factors (substrate concentration) but not through intrinsic factors, where finding a sweet spot in the design space of key genetic components (in this case, exogenous glycoside hydrolases) was needed.

## Supplementary Material

Buryskova_et_al_SI_2025_OUP_SynBio_ysaf012

## Data Availability

Source data to all displayed items are available at DOI: 10.5281/zenodo.15649047. All sequencing data are available under NCBI BioProject PRJNA1172556. Raw sequencing is available at the SRA database under accession numbers SRR31033117 (CP-Xpre) and SRR31033118 (CP-X). The whole-genome sequence of strain CP-X(pre) is available in the GenBank database under accession number CP172071. The code of the consortium model is available at https://github.com/BiocomputationLab/consortium. Fluorescence microscopy images are available in the Cell Image Library under accession number CIL:57521–57 527.
