# Supplementary material for: Construction of a syntrophic Pseudomonas putida consortium with reciprocal substrate processing
Source: Synth Biol (Oxf). 2025 Jun 24;10(1):ysaf012. doi: 10.1093/synbio/ysaf012 (PMC12341930; doi:10.1093/synbio/ysaf012)
Supplement: Buryskova_et_al_SI_2025_OUP_SynBio_ysaf012 [file buryskova_et_al_si_2025_oup_synbio_ysaf012.docx]

## **Supplementary information**

## **Construction of a syntrophic *Pseudomonas putida* consortium with reciprocal substrate processing**

Barbora Burýšková^1^, Jesús Miró-Bueno^2^, Barbora Popelářová^1^, Barbora Gavendová^1^, Ángel Goñi-Moreno^2^, Pavel Dvořák^1,*^

^1^ Department of Experimental Biology (Section of Microbiology), Faculty of Science, Masaryk University, Kamenice 753/5, 62500, Brno, Czech Republic

^2^ Systems Biology Department, Centro Nacional de Biotecnología, CSIC, Darwin 3, 28049, Madrid, Spain

***Corresponding author:**

Pavel Dvořák, Assoc. Prof.

Department of Experimental Biology (Section of Microbiology), Faculty of Science

Masaryk University, Kamenice 735/5, Brno 62500, Czech Republic

Phone: +420 549 493 396, E-mail: pdvorak@sci.muni.cz, https://orcid.org/0000-0002-3215-4763

**Supplementary tables**

**Supplementary Table 1.** List of strains used and prepared in this study.

| Strain | Characteristic | Reference |
| --- | --- | --- |
| *E. coli* |  |  |
| CC118 | *araD139 Δ(ara-leu)7697 ΔlacX74 phoAΔ20 galE galK thi rpsE rpoB argEam recAl* | ^1^ |
| DH5α λπ | DH5α lysogenised with λpir phage | Pablo Nikel´s Lab collection |
| DH5α λπ_pTNS-1 | Amp^R^, *ori* R6K, *tnsABCD*, strain with mobilisable helper plasmid | ^2^ |
| HB101_pRK600 | Chl^R^, *ori* ColE1, *tra*^+^ *mob*^+^ of RK2, helper strain | ^3^ |
| *P.putida* |  |  |
| EM42 | KT2440 derivative; Δprophage1-4 ΔTn*7* Δ*endA-1* Δ*endA-2* Δ*hsdRMS* Δflagellum ΔTn*4652* | ^4^ |
| Δ*gcd* | Δ*gcd* (glucose dehydrogenase PP_1444); chassis for the hexose eating strain | ^5^ |
| ID18 | Δ*gcd*::*mScarlet;* glycoside hydrolase^-^ control for CP-G | This work |
| CP-G | Δ*gcd*::*mScarlet* pSEVA2213_*xyl43A;* Hexose eater | This work |
| Δ*gcd::bglC* | Tn5 inserted *bglC* gene from *T. fusca* (P_em7_, N-His *bglC*, Sm^R^); reverse strand in *xerD* (PP_1468) | ^5^ |
| Δ*gcd/*Δ*glk::bglC* | Δ*glk* (glucokinase PP_1011); chassis for the pentose eating strain unable to consume glucose | This work |
| ID24 | Δ*gcd*/Δ*glk* pSW-I; glycoside hydrolase^-^ control for CP-X | This work |
| CP-X(pre) | Δ*gcd*/Δ*glk*::*bglC*/*msfGFP* pSEVA2213_*xylABE* | This work |
| CP-X | Δ*gcd*/Δ*glk*::*bglC*/*msfGFP* pSEVA2213_*xylABE;* Pentose eater; CP-X(pre) adapted on xylose; ~275 kbp multiplication | This work |
| CP-X_ssrA | CP-X with ssrA degradation tagged *bglC* | This work |
| CP-X_ssrActrl | CP-X with ssrActrl degradation tagged *bglC* | This work |
| CP-X_6A | CP-X with 6A degradation tagged *bglC* | This work |
| CP-X-NHis400 | CP-X, *bglC* without N-His tag + synthetic RBS (theoretical translation rate 400 a.u.) | This work |
| CP-X-NHis2500 | CP-X, *bglC* without N-His tag (theoretical translation rate 2,500 a.u.) | This work |
| CP-X-NHis2500_6A | CP-X_6A, *bglC* without N-His tag (theoretical translation rate 2,500 a.u.) | This work |

**Supplementary Table 2.** List of plasmids used and prepared in this study.

| Plasmid | Characteristics | Reference |
| --- | --- | --- |
| pSEVA2213 | Km^R^, *ori* RK2, P_em7_ | ^6^ |
| pSEVA238 | Km^R^, *ori* pBBR1, XylS/Pm | ^6^ |
| pSEVA2213_*xylABE* | Km^R^, *xylABE* genes from *E. coli* | ^5^ |
| pSEVA2213_*xyl43A*NHis | Km^R^, *xyl43A* gene from *T. fusca* | This work |
| pEMG _Δ*glk*HR | Km^R^, *ori* R6K, HR spanning (~500 bp) upstream and downstream of glucokinase (PP_1011) in *P. putida* KT2440 genome | Alberto Sánchez-Pascuala, CNB-CSIC |
| pSW-I | Amp^R^, *ori* RK2, XylS/Pm→ *I-sceI;* conditional I-SceI endonuclease expression | SEVA collection |
| pMRE-Tn7-145 | Amp^R^ Chl^R^ Gm^R^, mScarlet-I | ^7^ |
| pBG13 | Km^R^ Gm^R^, ori R6K, Tn*7*L and Tn*7*R extremes, P_em7_, BCD2-msfGFP fusion | ^8^ |
| pBG13_mScarlet | Km^R^ Gm^R^, ori R6K, Tn*7*L and Tn*7*R extremes, P_em7_, BCD2-mScarlet-I fusion, mScarlet-I N’(MVSK > MIMGILSK) | This work |
| pQURE1L | Amp^R^, *ori* RK2, XylS/Pm→*trfA,* XylS/Pm→*I-sceI,* P_14g_, mCherry | ^9^ |
| pSNW5 | Tet^R^, *traJ*, oriT, *ori* R6K, P_14g_, BCD2-msfGFP | ^9^ |
| pSNW5_*bglC*-HR | pSNW5 with HR spanning (~500 bp) upstream and downstream of *bglC* 3’ - end in CP-X genome | This work |
| pSNW5_*bglC*-HR_ssrA | pSNW5_*bglC*-HR with AANDENYALAA(*) encoding the ssrA degradation tag | This work |
| pSNW5_*bglC*-HR_ssrActrl | pSNW5_*bglC*-HR with AANDENYALDD(*) encoding the ssrActrl degradation tag | This work |
| pSNW5_*bglC*-HR_6A | pSNW5_*bglC*-HR with AAAAAA(*) encoding the 6A degradation tag | This work |
| pSNW5_*bglC*-HRw | pSNW5 with HR spanning (~500 bp) upstream and downstream of *bglC*, including the whole gene in CP-X genome | This work |
| pSNW5_*bglC*-HRw-NHis2500 | pSNW5_*bglC*-HRw without N-His (theoretical translation rate 2,500 a.u.) | This work |
| pSNW5_bglC-HRw-NHis400 | pSNW5_*bglC*-HRw without N-His, + synthetic RBS (theoretical translation rate 400 a.u.) | This work |

Abbreviations: Antibiotic markers – Amp Ampicillin, Gm Gentamicin, Chl Chloramphenicol, Km Kanamycin, Tet Tetracycline; HR Homology Region.

**Supplementary Table 3.** Primers used in this study. Annealing regions of the primers are highlighted in bold, restriction sites are underlined.

| Primer name | Sequence (5’→3’) | Used for |
| --- | --- | --- |
| glk del check fw | **gttgcggatcatggtgtcg** | Δ*glk* confirmation |
| glk del check rv | **tcgtcgaccatcaggatcg** |  |
| Xyl43A_N'His_BglII_fw | ataagatct**atgcaccatcaccatcac** | Amplification and restriction cloning of *xyl43A* from *T. fusca* into pSEVA2213. |
| Xyl43A_no-tag_XhoI_rv | tttctcgag**ggagggggactgagg** |  |
| pBG-Scarlet_fw | aatgaattc**tgagcaagggcgag** | Cloning mScarlet gene from pMRE-Tn7-145 to pBG13 to construct pBG13_mScarlet; uses *EcoR*I and changes beginning of protein sequence from MVSK to MIMGILSK. |
| pBG-Scarlet_rv | attgaatt**cttacttgtacagctcgtc** |  |
| bglCcpx_HR_24bp_fw | gctcggtacccggggatcctctag**gctgggtgtcaggcaacc** | Amplification and *in vivo* cloning of *bglC* region of CP-X strain into pSNW5 vector; overhangs homologous to vector sequence. |
| bglCcpx_HR_24bp_rv | agcttgcatgcctgcaggtcgact**ctctctccaggggaagccg** |  |
| pSNW_bglCHR_ssrAtag_fw | gccgctaacgacgagaactacgccctggctgcg**tagctgcaggcatgcaagc** | Amplification of pSNW5_*bglC*-HR vector, adding overhangs introducing ssrA degradation tag, *in vivo* cloning. |
| pSNW_bglCHR_ssrAtag_rv | cgcagccagggcgtagttctcgtcgttagcggc**ttcctgtccgaagattccccc** |  |
| pSNW_bglCHR_ssrAtagcntrl_fw | gccgctaacgacgagaactacgccctggatgac**tagctgcaggcatgcaagc** | Amplification of pSNW5_*bglC*-HR vector, adding overhangs introducing ssrActrl degradation tag, *in vivo* cloning. |
| pSNW_bglCHR_ssrAtagcntrl_rv | gtcatccagggcgtagttctcgtcgttagcggc**ttcctgtccgaagattccccc** |  |
| pSNW_bglCHR_6Atag_fw | gccgccgcagcagcagcg**tagctgcaggcatgcaagc** | Amplification of pSNW5_*bglC*-HR vector, adding overhangs introducing 6A degradation tag, *in vivo* cloning. |
| pSNW_bglCHR_6Atag_rv | cgctgctgctgcggcggc**ttcctgtccgaagattccccc** |  |
| SZ3 | **gctgcgttcggtcaaggttc** | Sequencing^8^. |
| tagcheck_fw | **tggtactcccgggtgatgc** | Used with SZ3 to check for tag presence after integration and HR repair (132bp - /150bp 6A/165bp ssrA). |
| xerD_joint | aatctgaattcgagctcg**ccagcagcatctgtaccacc** | Amplification of *bglC* upstream part in CP-X, removing N-His and adjusting RBS to 400 a.u., *in vivo* cloning. |
| bglC400_rv | cattatttcaggattaccgagatatgagtgtt**ggtttagttcctcaccttgtcg** |  |
| bglC400_fw | aacactcatatctcggtaatcctgaaata**atgacctcgcaatcgacgactc** | Amplification of *bglC* front part in CP-X, removing N-His and adjusting RBS to 400 a.u., *in vivo* cloning. |
| bglCfront_rv | gttgcctgacacccagc**tcgggttgtagaagttgacgc** |  |
| bglC-His_fw | attcgagctcggtaccctttaagaaggagatatacat**atgacctcgcaatcgacg** | Amplification of pSNW5_*bglC*-HRw-NHis400, overhangs adjusting RBS to 2500 a.u., *in vivo* cloning. |
| bglC-NHis_rv | gtaccgagctcgaattcgcgcgcggcggcctacg**ggtttagttcctcaccttgtcg** |  |
| bglC-Nterm_rv | **atctggaacgaagcggtcg** | Check for N-terminus change after integration and HR repair. |
| pEMG_opt_fw | **tcttcgctattacgccagc** | Sequencing checks. |
| pEMG_seq_rv | **ctttacactttatgcttccggc** |  |
| pEM7_fw | **gttgttgacaattaatcatcggcatag** |  |
| bglC seq 1 | **actacgcggagatcgtctac** |  |
| bglC seq 2 | **cttcgaggacaaggtggtcg** |  |

**Supplementary Table 4.** Presence of a genomic multiplication in the chromosome of strain CP-X.

| **Coverage** | | | | | |
| --- | --- | --- | --- | --- | --- |
|  | **Chromosome without multiplied region (C)** | | **Multiplied region (M)** | | **M/C ratio** |
| **Strain** | **Nanopore reads** | **SD** | **Nanopore reads** | **SD** |  |
| **CP-X** | 98.3 | 12.8 | 250.7 | 25.8 | **2.6** |
| **CP-X(pre)** | 77.4 | 11.5 | 77.2 | 6.9 | **1** |

Coverage was determined by the Geneious algorithm (Geneious Prime 2024.0.5). 275-kb region bordered by IS elements in pos. 2,220,489 - 2,495,683 bp in the reference genome of CP-X(pre).

**Supplementary figures**


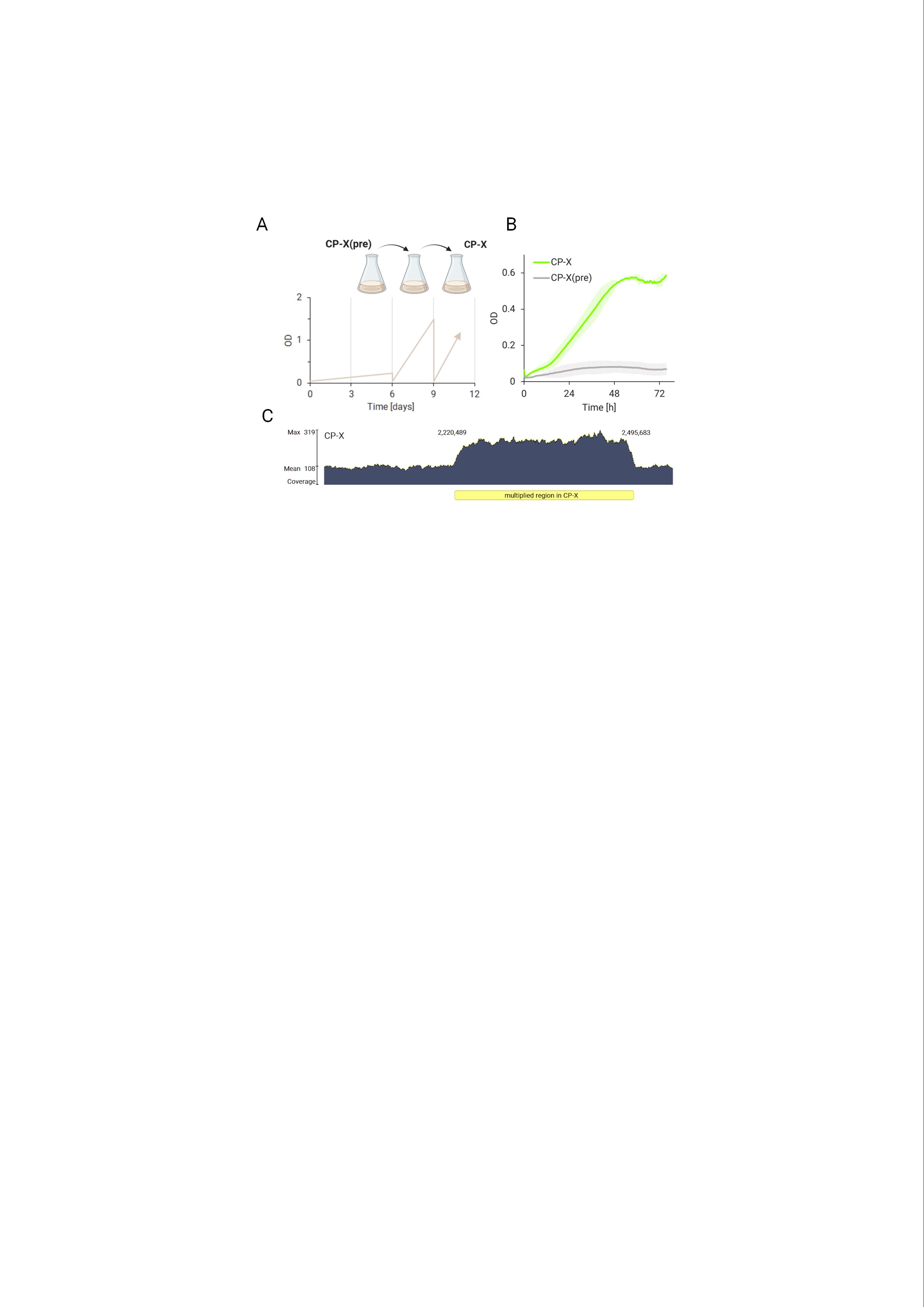
**Supplementary Figure 1**. Xylose eater adaptation on xylose. (**A**) Reinoculation of the template strain CP-X(pre) in M9 minimal medium with 5 g/l of xylose as the sole carbon source led to an improved phenotype (CP-X). (**B**) Growth of both strains was compared in a 96-well plate cultivation on M9 medium with 2 g/l xylose. Data are shown as means ± standard deviations from two biological replicates (n = 2). (**C**) Sequencing revealed the emergence of a large genomic multiplication in the adapted strain CP-X.

**
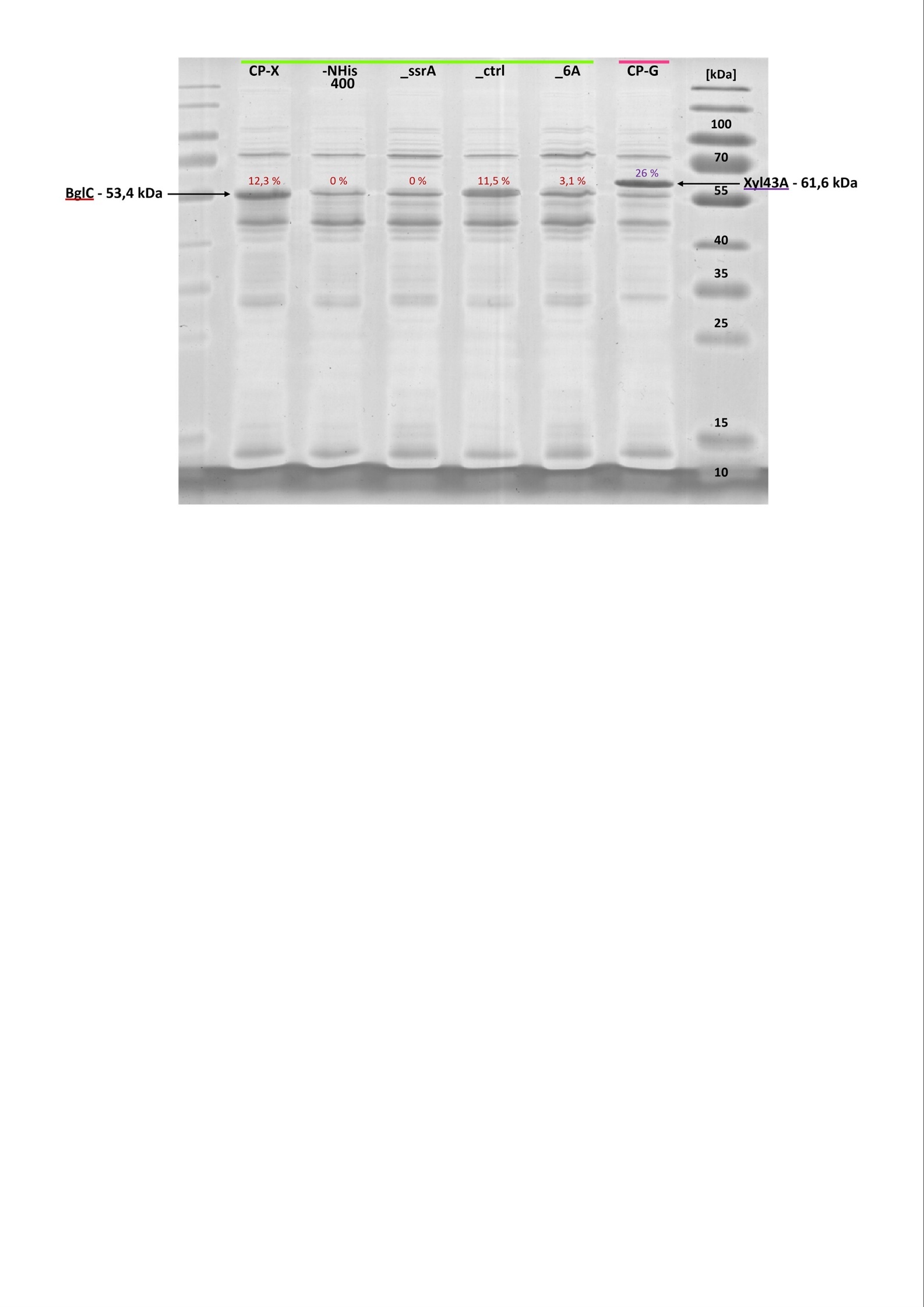
Supplementary Figure 2**. SDS-PAGE of consortium strain cell-free extracts. A 12% polyacrylamide (acrylamide and bis-acrylamide solution, 37.5:1) separating gel was used. Samples from the left: CP-X, CP-X-NHis400, CP-X_ssrA, CP-X_ssrActrl, CP-X_6A, CP-G. Protein abbreviations: BglC β-glucosidase, Xyl43A β-xylosidase.


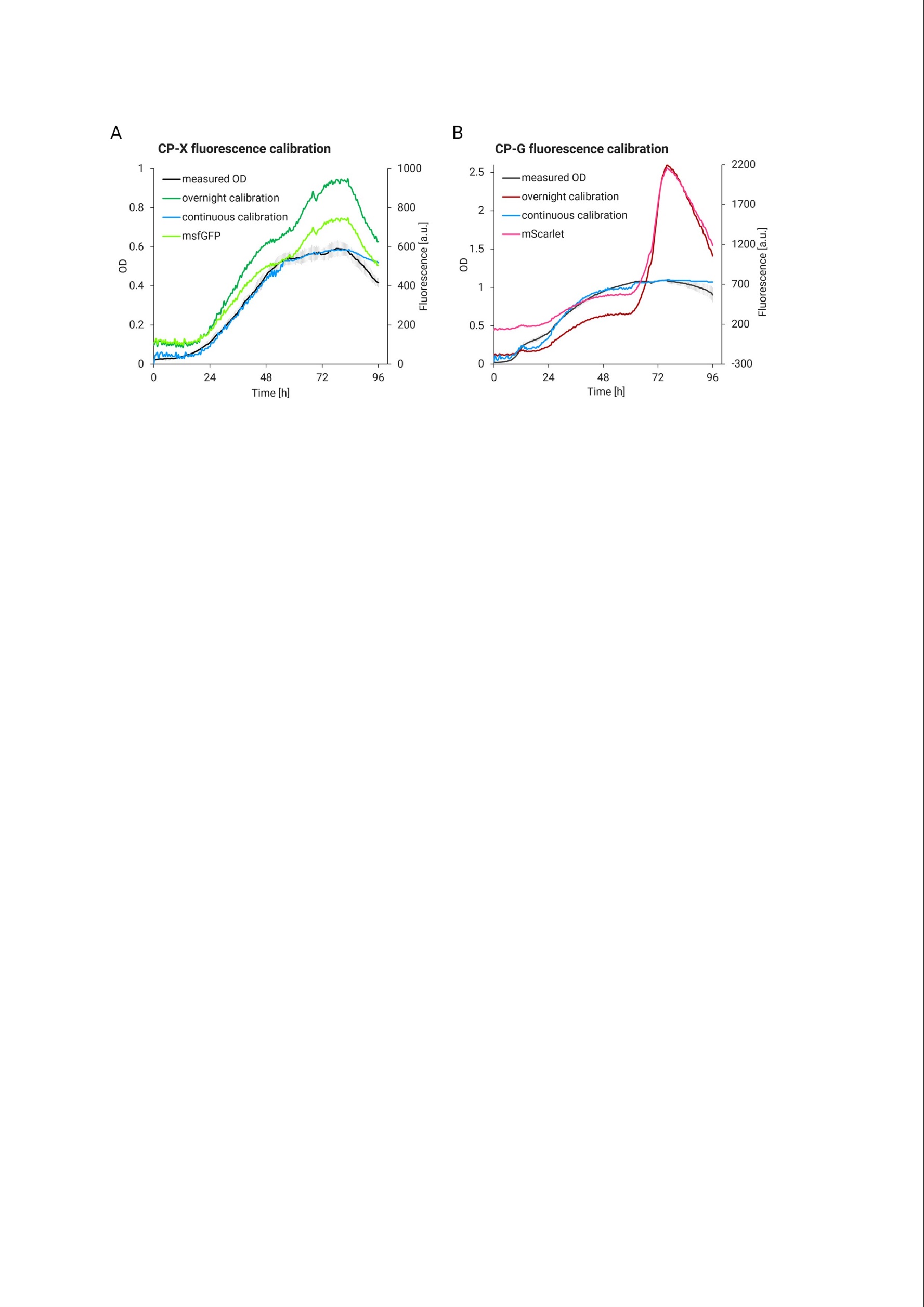


**Supplementary Figure 3**. Comparison of fluorescence calibration options for the 96 well-plate cocultivation format. Overnight calibration was done by serial dilution of cells grown on rich LB medium to stationary phase. Continuous calibration was done by measuring fluorescence and OD in real-time during growth in M9 minimal medium with monosaccharides (2 g/l). Data are shown as means from four biological replicates (n = 4). Error bars except for measured OD are omitted for clarity.

**Supplementary Figure 4**. Absolute growth rate (dOD/dt) patterns of consortium strains. (**A**) CP-X absolute growth rate pattern in monoculture (from Fig.3B left; xylose 2 g/l) and in cooperation with CP-G (from Fig.3C right; cellobiose 1 g/l + xylobiose 1 g/l). (**B**) CP-G absolute growth rate pattern in monoculture (from Fig.3B right; glucose 2 g/l) and in cooperation with CP-X (from Fig.3C right; cellobiose 1 g/l + xylobiose 1 g/l). (**C**) CP-X and CP-G absolute growth rate patterns in cooperation (from Fig.3C right; cellobiose 1 g/l + xylobiose 1 g/l). Absolute growth rates were calculated using the numpy.gradient function from the NumPy library in Python, with the experimental OD data as the input. The input data was first smoothed using the savgol_filter function from the scipy.signal library in Python
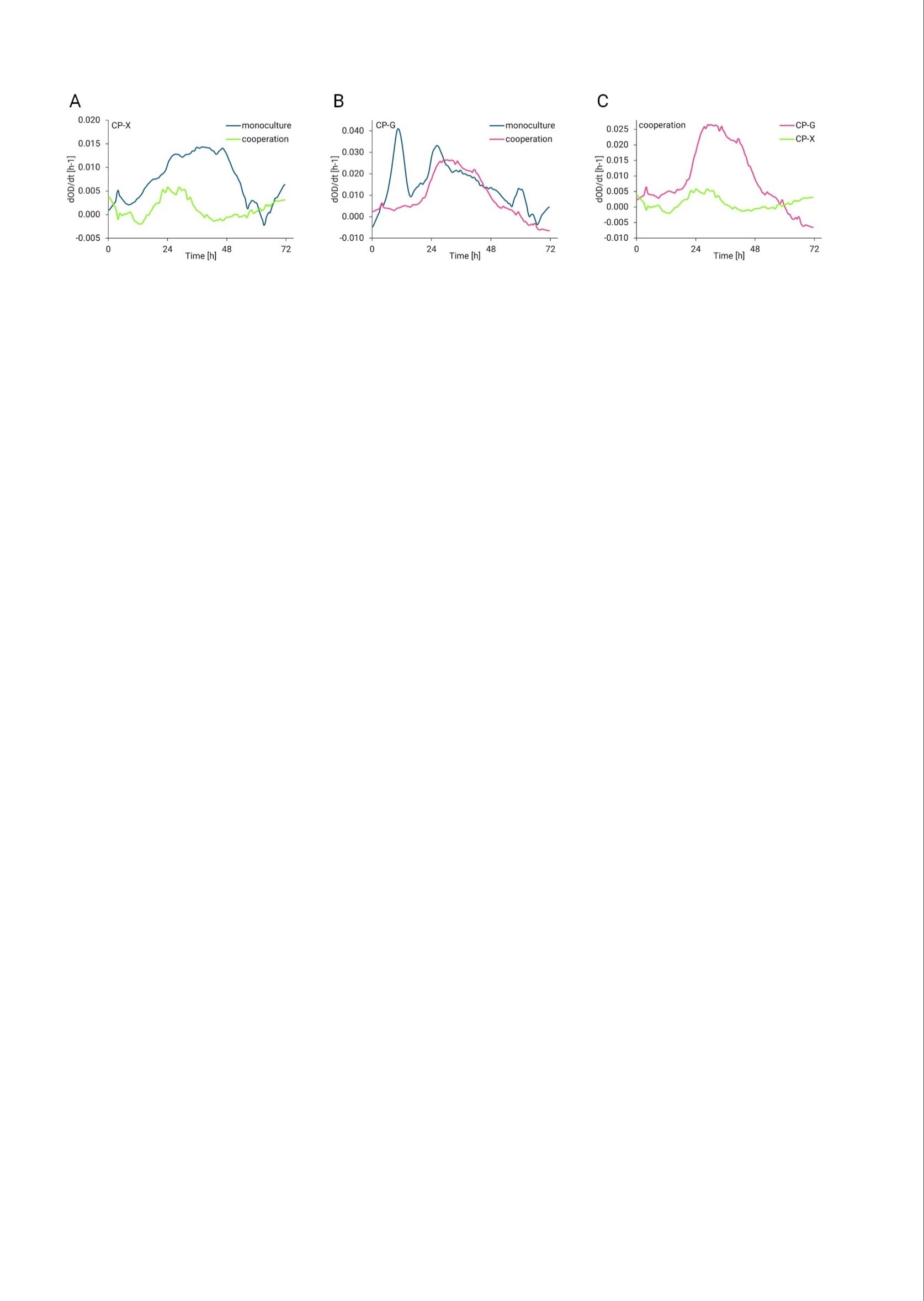
.


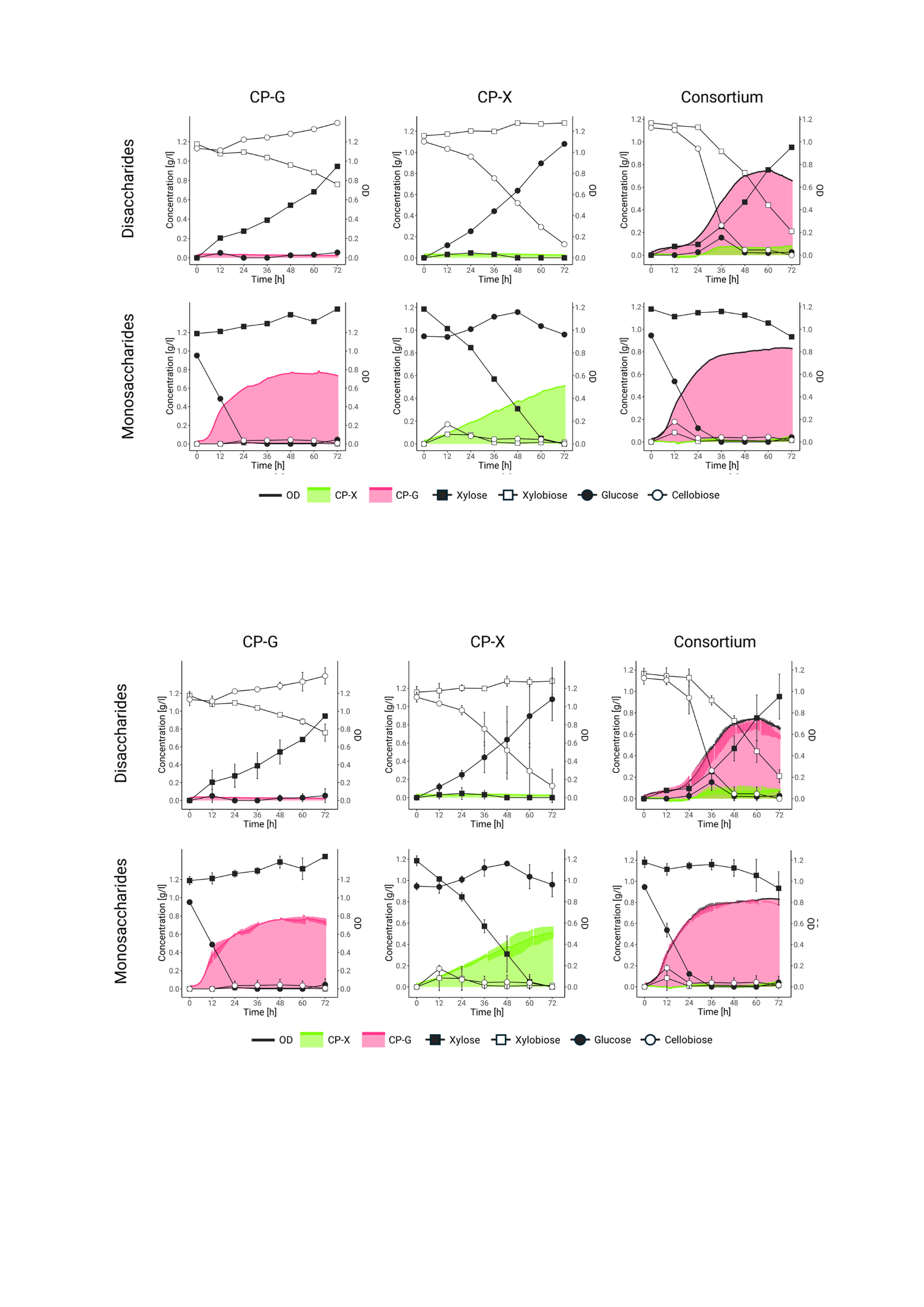


**Supplementary Figure 5**. Consortium and monoculture cultivations on disaccharides and monosaccharides in 96-well plate format with saccharide concentration monitoring by HPLC. OD is depicted as an area mapped on the right y-axis, whereas sugar concentrations are depicted with line graphs mapped on the left y-axis. Data are shown as means of at least three biological replicates from two independent experiments (n ≥ 3). Note that slight evaporation of culture volume towards the end of the experiment causes sample concentration, which makes the amount of abundant sugars seem to rise.

**Supplementary Figure 6**. Cell cluster formation in the tightly linked consortium of CP-G and CP-X_ssrA. On the left, a micrograph of cell clusters recorded with ZEISS Elyra 7. Consortium of CP-G tagged by mScarlet and CP-X_ssrA tagged by msfGFP cooperating at growth on disaccharides. Photo taken at the end of cultivation (72 h). The table on the right reports the amounts of cell clusters formed during the growth in stress conditions (minimal medium without carbon source) and cooperative growth on disaccharides. The experiment was conducted in two biological replicates (n = 2).


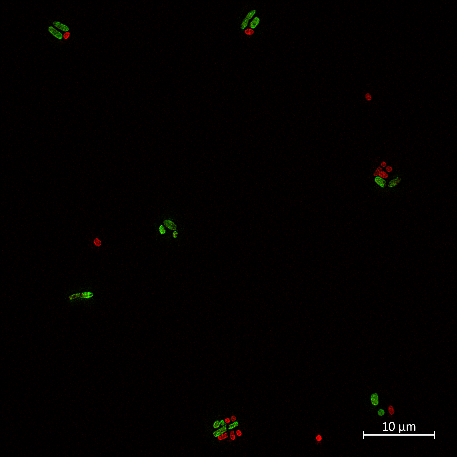

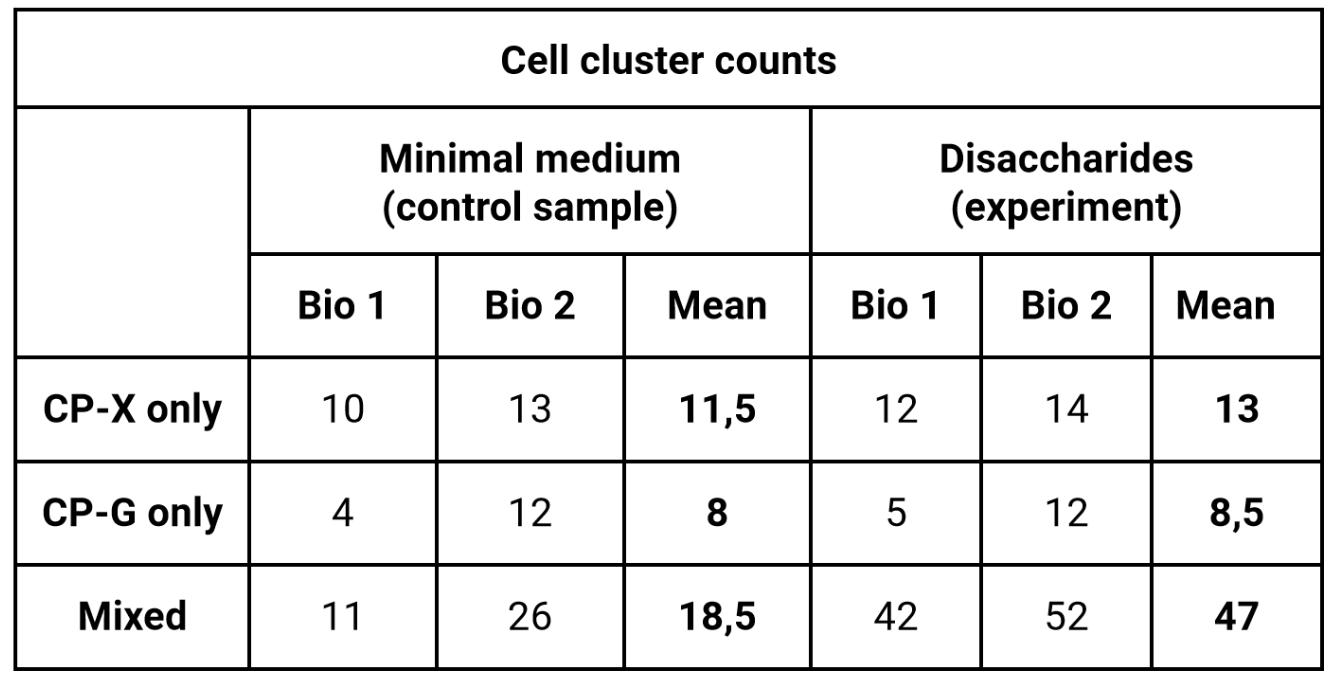


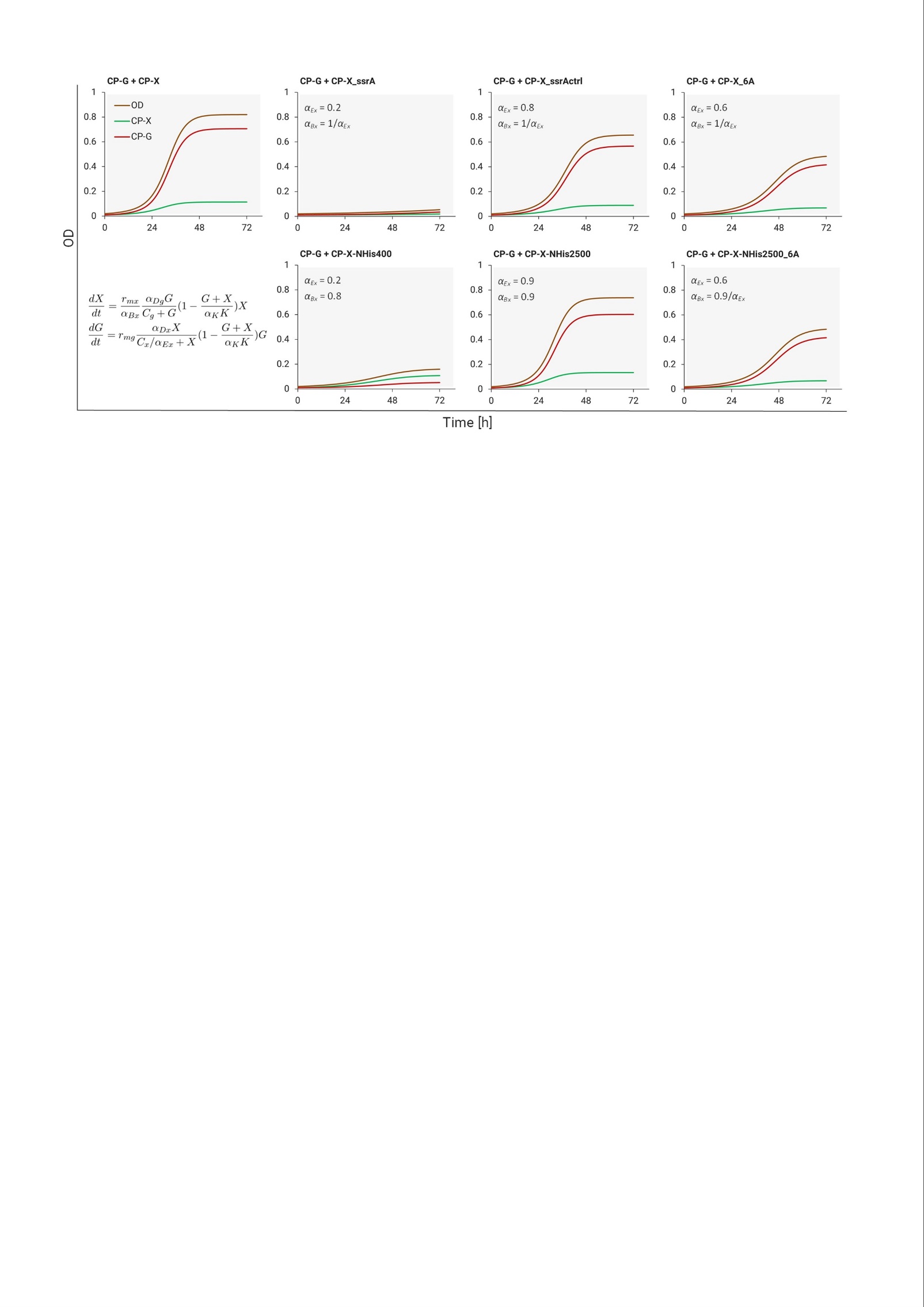


**Supplementary Figure 7**. Mathematical model simulations of cooperating consortium strains with modulated levels of BglC expression and degradation. For the ctrl, 6A, ssrA, NHis400, NHis2500 and NHis2500_6A strains, the values of *α_Ex_* are 0.8, 0.6, 0.2, 0.2, 0.9 and 0.6, respectively. In ssrA, ctrl, and 6A strains, we assume the burden is inversely proportional to the amount of BglC, following the equation *α_Bx_* = 1/*α_Ex_*, to account for ATP-dependent BglC degradation. In NHis400 and NHis2500 strains, *α_Bx_* = 0.8 and 0.9, we assume *α_Bx_*<1 because the expression of BglC is lower, resulting in a decreased metabolic burden. For NHis2500_6A, we use *α_Bx_* = 0.9/*α_Ex_* (*α_Ex_* = 0.6) to account for both the lower amount of BglC and the burden caused by BglC targeting to ATP-dependent proteasomes.


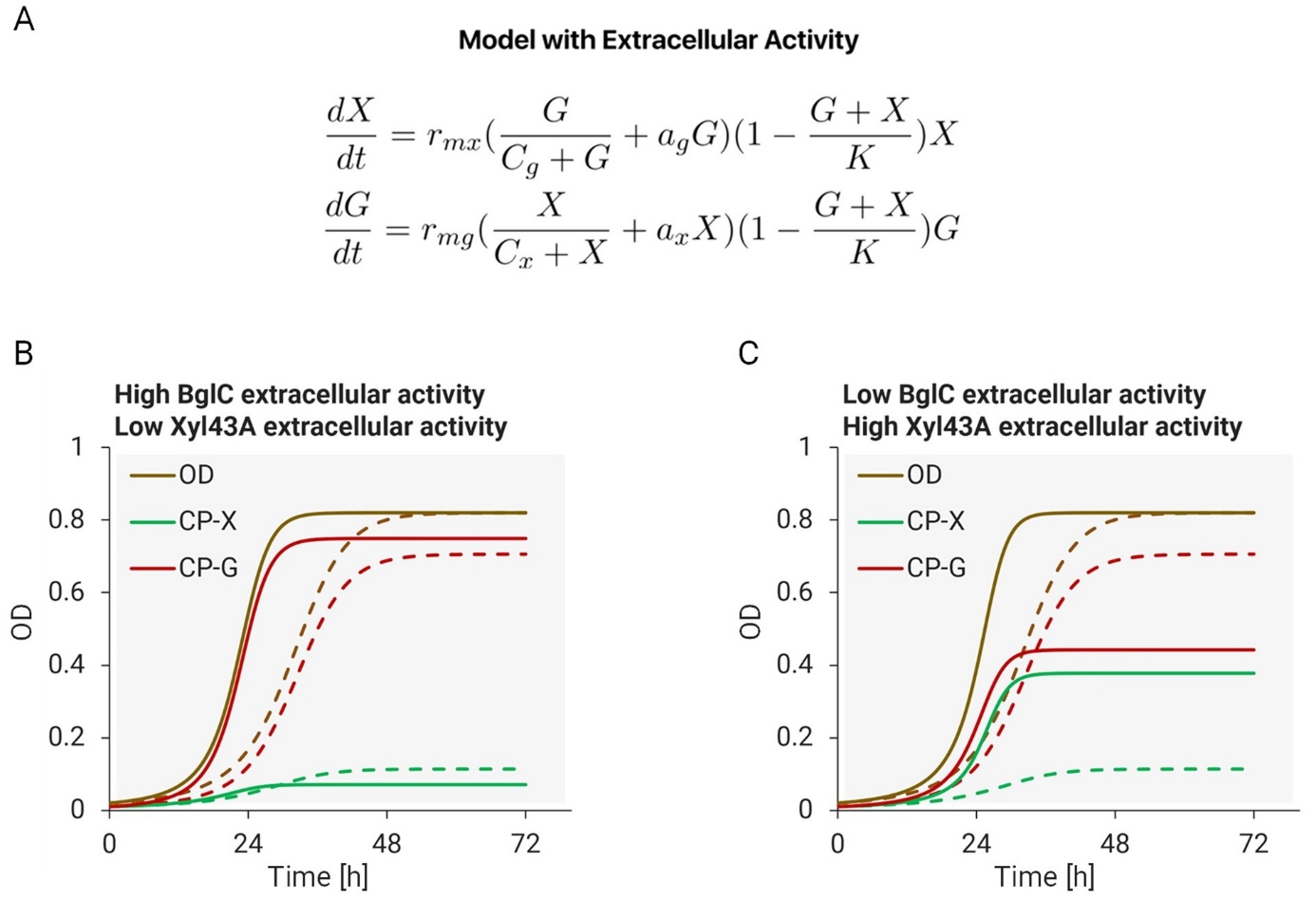


**Supplementary Figure 8**. Consortium model incorporating extracellular activity. (**A**) Differential equations describing the dynamics of the two-strain consortium (CP-X and CP-G). This model extends the one presented in Figure 3E in the main text by incorporating potential extracellular activity of BglC (from CP-X) and Xyl43A (from CP-G). The term a_x_X represents glucose generated by extracellular BglC activity, while the term a_g_G represents xylose generated by extracellular Xyl43A activity. (**B**) Simulation under conditions of high extracellular BglC activity and low extracellular Xyl43A activity (a_x_ = 10, a_g_ = 1; solid lines). (**C**) Simulation under conditions of low extracellular BglC activity and high extracellular Xyl43A activity (a_x_ = 1, a_g_ = 10; solid lines). All other parameters and initial conditions are identical to those in Figure 3E. The original model from Figure 3E is included for reference (dashed lines).

### **References**

1. Manoil, C., and Beckwith, J. (1985) TnphoA: a transposon probe for protein export signals. *Proc. Natl. Acad. Sci. U. S. A. 82*(23), 8129-8133.
2. Choi, K. H., Gaynor, J. B., White, K. G., Lopez, C., Bosio, C. M., Karkhoff-Schweizer, R. R., and Schweizer, H. P. (2005) A Tn 7-based broad-range bacterial cloning and expression system. *Nat. Methods*. *2*(6), 443-448.
3. Boyer, H. W., and Roulland-Dussoix, D. (1969) A complementation analysis of the restriction and modification of DNA in *Escherichia coli*. *J. Mol. Biol.* *41*(3), 459-472.
4. Martínez-García, E., Nikel, P. I., Aparicio, T., and de Lorenzo, V. (2014) Pseudomonas 2.0: genetic upgrading of *P. putida* KT2440 as an enhanced host for heterologous gene expression. *Microb. Cell Fact.* *13*, 159.
5. Dvořák, P., and de Lorenzo, V. (2018) Refactoring the upper sugar metabolism of *Pseudomonas putida* for co-utilization of cellobiose, xylose, and glucose. *Metab. Eng.* *48*, 94-108.
6. Silva-Rocha, R., Martínez-García, E., Calles, B., Chavarría, M., Arce-Rodríguez, A., de Las Heras, A., and De Lorenzo, V. (2013) The Standard European Vector Architecture (SEVA): a coherent platform for the analysis and deployment of complex prokaryotic phenotypes. *Nucleic Acids Res.* *41*(D1), D666-D675.
7. Schlechter, R. O., Jun, H., Bernach, M., Oso, S., Boyd, E., Muñoz-Lintz, D. A., and Remus-Emsermann, M. N. (2018) Chromatic bacteria–A broad host-range plasmid and chromosomal insertion toolbox for fluorescent protein expression in bacteria. *Front. Microbiol*. *9*, 3052.
8. Zobel, S., Benedetti, I., Eisenbach, L., de Lorenzo, V., Wierckx, N., and Blank, L. M. (2015) Tn7-based device for calibrated heterologous gene expression in *Pseudomonas putida*. *ACS Synth. Biol.* *4*(12), 1341-1351.
9. Volke, D. C., Friis, L., Wirth, N. T., Turlin, J., and Nikel, P. I. (2020) Synthetic control of plasmid replication enables target-and self-curing of vectors and expedites genome engineering of *Pseudomonas putida*. *Metab. Eng. Commun. 10*, e00126.
